# GLP‐1 Receptor Agonists Alleviate Diabetic Kidney Injury via *β*‐Klotho‐Mediated Ferroptosis Inhibition

**DOI:** 10.1002/advs.202409781

**Published:** 2024-12-04

**Authors:** Shasha Tian, Saijun Zhou, Weixi Wu, Yao lin, Tongdan Wang, Haizhen Sun, A‐Shan‐Jiang A‐Ni‐Wan, Yaru Li, Chongyang Wang, Xiaogang Li, Pei Yu, Yanjun Zhao

**Affiliations:** ^1^ NHC Key Laboratory of Hormones and Development, Chu Hsien‐I Memorial Hospital and Tianjin Institute of Endocrinology, Tianjin Key Laboratory of Metabolic Diseases Tianjin Medical University Tianjin 300134 China; ^2^ Department of Nephrology The Fifth Hospital of Shanxi Medical University (Shanxi Provincial People's Hospital) Taiyuan Shanxi 030000 China; ^3^ School of Pharmaceutical Science & Technology, Tianjin Key Laboratory for Modern Drug Delivery & High Efficiency, Faculty of Medicine Tianjin University Tianjin 300072 China; ^4^ School of Life Sciences Peking University Beijing 100871 China; ^5^ Department of Internal Medicine Mayo Clinic Rochester MN 55901 USA; ^6^ Nephropathy & Blood Purification Department The Second Hospital of Tianjin Medical University Tianjin 300134 China

**Keywords:** diabetic kidney disease, ferroptosis, GLP‐1 receptor agonist, semaglutide, β‐Klotho

## Abstract

Semaglutide (Smg), a GLP‐1 receptor agonist (GLP‐1RA), shows renal protective effects in patients with diabetic kidney disease (DKD). However, the exact underlying mechanism remains elusive. This study employs transcriptome sequencing and identifies *β*‐Klotho (KLB) as the critical target responsible for the role of Smg in kidney protection. Smg treatment alleviates diabetic kidney injury by inhibiting ferroptosis in patients, animal models, and HK‐2 cells. Notably, Smg treatment significantly increases the mRNA expression of KLB through the activation of the cyclic adenosine monophosphate (cAMP) signaling pathway, specifically through the phosphorylation of protein kinase A (PKA) and cAMP‐response element‐binding protein (CREB). Subsequently, the adenosine monophosphate‐activated protein kinase (AMPK) signaling pathway is activated, reprograming the key metabolic processes of ferroptosis such as iron metabolism, fatty acid synthesis, and the antioxidant response against lipid peroxidation. Suppression of ferroptosis by Smg further attenuates renal inflammation and fibrosis. This work highlights the potential of GLP‐1RAs and KLB targeting as promising therapeutic approaches for DKD management.

## Graphical abstract

Semaglutide (Smg), a GLP‐1 receptor agonist (GLP‐1RA), boosts the *β*‐klotho (KLB) expression in renal tubular epithelial cells under the challenge of high levels of glucose and lipid. The upregulation of KLB further modulates the lipid metabolism, iron metabolism, and lipid peroxidation, resulting in the inhibition of ferroptosis, and the attenuation of renal inflammation and fibrosis to relieve the diabetic kidney disease (DKD).

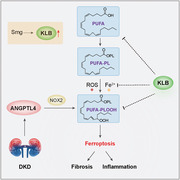

## 1 Introduction

Diabetic kidney disease (DKD) is a significant complication of diabetes, initiated and exacerbated by hyperglycemia, mitochondrial dysfunction, oxidative stress, and inflammation. It stands as the predominant cause of end‐stage renal diseases,^[^ ^1^, ^2^ ^]^ and tubulointerstitial fibrosis is one of the key manifestations involved in this progression.^[^ ^3^ ^]^ Glucagon‐like peptide‐1 (GLP‐1) is an endogenous human hormone that binds to the GLP‐1 receptor, modulating pancreatic islet cell function to potentiate insulin secretion.^[^ ^4^ ^]^ The introduction of GLP‐1 receptor agonists (GLP‐1RAs) has revolutionized the management of type 2 diabetes, which is often associated with nephropathy and other adverse complications.^[^ ^5^ ^]^ Mounting evidence indicates that treatment with GLP‐1RAs can improve kidney function beyond merely lowering glucose levels in patients with DKD.^[^ ^6^, ^7^ ^]^ However, the mechanism underlying the renal protective effects of GLP‐1RAs remains elusive. Analysis of kidney biopsy samples from 28 DKD patients revealed a decrease in the expression of mRNAs for both glutathione peroxidase 4 (GPX4) and solute carrier family 7 member 11 (SLC7A11) in the kidney tubules of DKD patients compared to those in healthy individuals.^[^ ^8^ ^]^ GPX4 and SLC7A11 are critical modulators of ferroptotic cell death,^[^ ^9^ ^]^ and thus we postulated that ferroptosis might play a role in the renal protective effects mediated by GLP‐1RAs.

Ferroptosis is an iron‐dependent form of regulated cell death that was discovered in 2012.^[^ ^10^ ^]^ The hallmarks of ferroptosis include redox‐active iron (Fe^2+^), oxidized arachidonic/adrenic phosphatidylethanolamines (PE‐AA‐OOH/PE‐AdA‐OOH), and insufficient lipid peroxide repair.^[^ ^11^ ^]^ GPX4, a key regulator of ferroptosis, along with its cofactor glutathione (GSH), converts harmful phospholipid hydroperoxides into non‐toxic lipid alcohols.^[^ ^12^, ^13^ ^]^ SLC7A11 is responsible for the import of cystine and the biosynthesis of GSH. Besides, coenzyme Q_10_ functions as a key radical‐trapping antioxidant to suppress lipid peroxidation, with its recycling involving ferroptosis suppressor protein 1 (FSP1) and dihydroorotate dehydrogenase.^[^ ^14^, ^15^, ^16^ ^]^ Tetrahydrobiopterin serves as another radical‐trapping agent, with its biosynthesis and recycling dependent on GTP cyclohydrolase‐1 and dihydrofolate reductase, respectively.^[^ ^17^ ^]^ Moreover, the membrane‐bound O‐acyltransferase domains containing 1 and 2 impede the generation of tailored ferroptotic phospholipids through lipid remodeling.^[^ ^18^ ^]^ Renal tubular cells are particularly susceptible to ferroptosis, and extensive research has revealed an essential role of ferroptosis in the development of diabetic nephropathy.^[^ ^8^, ^19^, ^20^ ^]^

Studies have demonstrated that ferroptosis is involved in regulating cellular inflammation.^[^ ^21^ ^]^ Cells undergoing ferroptosis release substantial amounts of damage‐associated molecular patterns, which are signaling molecules that promote inflammatory responses and exacerbate kidney damage, leading to chronic fibrosis.^[^ ^22^ ^]^ Activation of various inflammation‐related pathways can also lead to the onset and progression of ferroptosis.^[^ ^23^ ^]^ Disturbed iron metabolism due to diabetic renal cell injury increases the risk of oxidative stress and inflammation, further exacerbating the kidney injury.^[^ ^24^ ^]^ Furthermore, evidence suggests that ferroptosis plays a pathogenic role in the transition from acute kidney injury to chronic kidney disease and in the development of renal fibrosis.^[^ ^25^ ^]^ Inhibiting ferroptosis has been shown to alleviate unilateral ureteral obstruction‐induced fibrosis in renal tubular epithelial cells and diabetes‐induced renal epithelial‐matrix transition.^[^ ^26^, ^27^ ^]^

We postulated that GLP‐1RAs might protect the structure and function of the kidney in diabetes patients via ferroptosis inhibition. To clarify the corresponding mechanism of action, we utilized semaglutide (Smg), one of the GLP‐1RAs, to treat proximal tubular cells (HK‐2 cells) under diabetic conditions. Transcriptomic analysis revealed that treatment with Smg significantly increased the expression of the gene β‐klotho (KLB) in HK‐2 cells. KLB serves as a primary receptor for fibroblast growth factor 21 (FGF21) in conjunction with fibroblast growth factor receptor (FGFR) to mediate glucose and lipid metabolism as well as energy expenditure; the loss of KLB negates all effects of FGF21.^[^ ^28^ ^]^ Previous research has shown that FGF21 can attenuate liver injury and fibrosis by inhibiting ferroptosis.^[^ ^29^ ^]^ KLB has been shown to promote β‐cell survival and insulin biosynthesis independently of FGF21.^[^ ^30^ ^]^ Thus, we hypothesized that KLB may independently inhibit ferroptosis, a role that has not been previously reported in renal tubular epithelial cells. We performed clinical trials and in vitro and in vivo experiments to investigate the critical role of KLB in ferroptosis inhibition. This work elucidates a novel molecular mechanism by which GLP‐1RAs may protect diabetic kidneys from ferroptosis‐mediated injury.

## 2 Results

### 2.1 Semaglutide Reduces Kidney Injury and Attenuates Ferroptosis in DKD Patients

To verify the effect of Smg on renal protection in DKD patients, we conducted a pilot study involving 28 DKD patients. Among them, 15 patients received insulin (Ins) treatment and were categorized as the control group (i.e., DKD/Ins), while another 13 patients received Smg treatment (i.e., DKD/Smg). The treatment period lasted for 28 weeks, after which we investigated the effect of Smg on kidney injury protection. We initially compared the baseline data of the two groups and found no significant difference (Table S1, Supporting Information). Subsequently, we assessed the changes in various measured indicators from baseline to the 28‐week follow‐up period in both groups. As shown in **Table** **1**, we observed statistically significant differences in the changes of HbA1c, waist‐to‐hip ratio, and indicators reflecting renal function (serum creatinine, eGFR, UACR, NAG, RBP, and TRF) between the two groups.

**Table 1:** Comparison of the change of clinical indicies in the two groups.

Parameters	Control [*n* = 15]	DKD/Smg [*n* = 13]	*P* value
	△value	△value	
HbA1c, %	−0.40 ± 0.67	−0.89 ± 0.69	**0.041**
Fasting blood glucose, mmol l^−1^	−1.53 ± 1.38	−1.77 ± 0.83	0.650
Bodyweight, kg	−1.28 ± 4.38	−5.91 ± 8.74	0.118
BMI, kg m^−2^	−0.66 ± 1.33	−2.13 ± 3.11	0.170
Waist‐to‐Hip Ratio	0.01 ± 0.07	−0.08 ± 0.11	**0.010**
Albumin, g l^−1^	−0.08 ± 2.43	−0.14 ± 4.73	0.717
Alanine transaminase, U l^−1^	0.11 ± 4.28	−0.77 ± 15.65	0.650
Aspartate transaminase, U l^−1^	1.03 ± 5.75	0.43 ± 6.13	0.751
Alkaline phosphatase, U l^−1^	−5.41 ± 18.97	−15.88 ± 35.71	0.316
Blood urea nitrogen, µmol l^−1^	0.86 ± 4.92	−0.22 ± 2.97	0.856
Serum creatinine, µmol l^−1^	3.25 ± 9.50	−11.78 ± 14.38	**0.002**
eGFR, mL min^−1^/1.73 m^2^	−1.97 ± 4.03	5.43 ± 6.20	**0.002**
Serum uric acid, µmol l^−1^	25.31 ± 70.68	−10.11 ± 162.09	0.363
Triglycerides, mmol l^−1^	−0.47 (−1.45, 0.86)	−0.53 (−0.84, 0.03)	0.928
Total cholesterol, mmol l^−1^	−0.24 ± 1.67	−0.33 ± 0.99	0.892
High‐density lipoprotein, mmol l^−1^	0.14 ± 0.41	−0.05 ± 0.21	0.156
Low density lipoprotein, mmol l^−1^	−0.30 ± 1.05	−0.19 ± 0.54	0.964
UACR, mg g^−1^	5.07 (−83.22, 590.54)	−114.86 (−1045.85, – 27.81)	**0.015**
NAG, U l^−1^	0.18 ± 6.92	−7.25 ± 10.38	**0.025**
RBP, mg l^−1^	0.17 (−1.92, 0.63)	−7.52 (−25.74, – 0.98)	**0.002**
TRF, mg l^−1^	0.17 (−5.16, 0.68)	−20.48 (−22.20, – 7.43)	**0.001**
β2MG, mg l^−1^	0.12 (−0.07, 0.99)	−0.32 (−1.66, 0.17)	0.142
IgG, mg l^−1^	−9.76 ± 19.86	−10.86 ± 18.45	0.274

Data are presented as mean ± standard error (*n* ≥13). Statistical comparison was performed using an unpaired two‐tailed Student's *t*‐test. *p* < 0.05 was considered statistically significant. eGFR: estimated glomerular filtration rate; HbA1c: Hemoglobin A1c; UACR: Urinary Albumin/Creatinine Ratio; NAG: N‐acetyl‐β‐D‐glucosaminidase; RBP: Retinol Binding Protein; TRF: Transferrin; β2MG: β2‐microglobulin.

**Figure 1:**
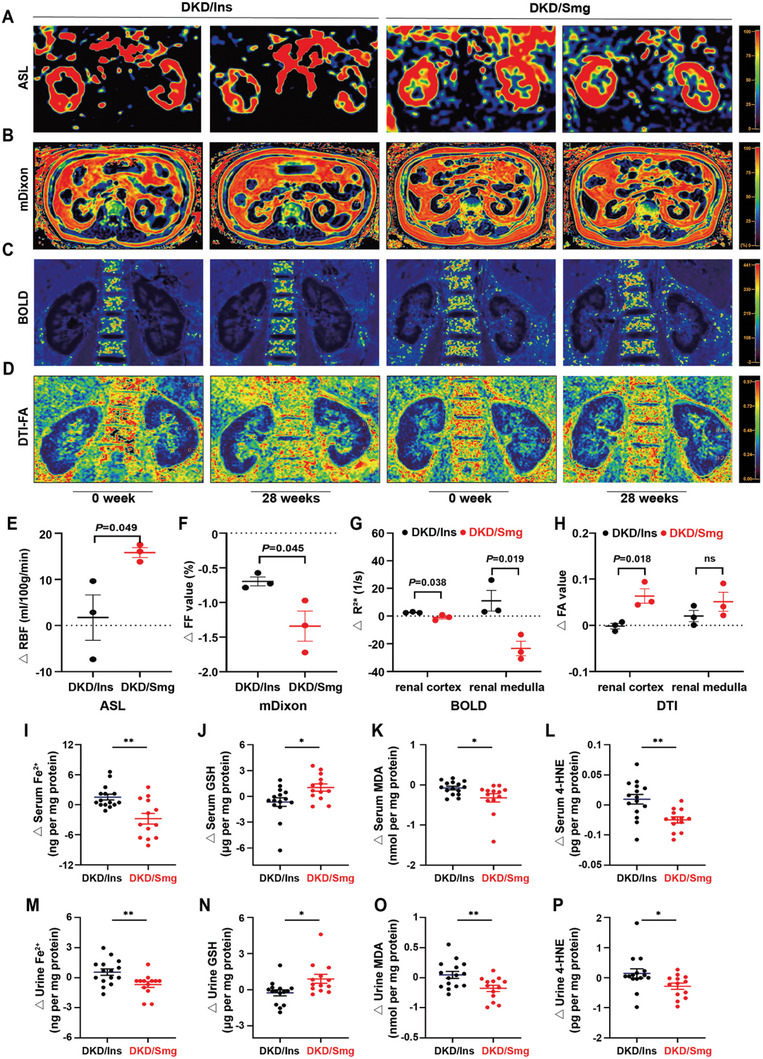
Semaglutide (Smg) protects the kidney and inhibits ferroptosis in patients with diabetic kidney disease (DKD). Representative magnetic resonance images of DKD patients receiving insulin detemir (once daily, DKD/Ins) or Smg (0.5 mg once weekly, DKD/Smg) at the timepoints of baseline and week 28: A–D) ASL, mDixon, BOLD, and DTI‐FA. E–H) Comparison of the change of RBF, FF, *R* ^2^*, and FA. I–L) The changes in serum levels of Fe^2+^, GSH, MDA, and 4‐HNE in DKD/Ins and DKD/Smg groups. M–P) The changes in urine levels of Fe^2+^, GSH, MDA, and 4‐HNE in the two groups. ASL: arterial spin labeling, mDixon: modified Dixon; BOLD: blood oxygenation level‐dependent; DTI‐FA: diffusion tensor imaging for fractional anisotropy; RBF: renal blood flow; FF: fat fraction; GSH: glutathione; MDA: malondialdehyde; 4‐HNE 4‐hydroxynonenal. Data are presented as mean ± standard error (A–D, *n* = 3; I–P, *n* ≥13). Statistical comparison was performed using an unpaired two‐tailed Student's *t*‐test. ns, no significant difference, *p < 0.05, **p < 0.01, ***p < 0.001.

Non‐invasive magnetic resonance imaging (MRI) was employed to assess changes in renal function in DKD patients undergoing Smg and Ins treatments. Initially, we utilized the arterial spin labeling (ASL) MRI approach to measure renal blood flow (RBF), using magnetically labeled arterial blood water as the endogenous tracer (**Figure** **1**A). Our results indicated a significant increase in RBF in DKD patients treated with Smg compared to the control group (Figure 1E). Using the mDixon MRI technique, which employs fat fraction (FF) as an index, we observed that Smg treatment largely suppressed fat accumulation in and around the kidneys of DKD patients (Figure 1B, F). The blood oxygenation level‐dependent (BOLD) MRI utilized deoxyhemoglobin as a contrast agent to assess tissue oxygenation (Figure 1C). Treatment with Smg substantially relieved hypoxia in both the renal cortex and medulla of DKD patients (Figure 1G). Furthermore, diffusion tensor imaging (DTI) MRI demonstrated that Smg treatment increased water fractional anisotropy (FA) in the renal cortex, while no significant changes were observed in the medulla (Figure 1D,H). Collectively, the MRI analyses strongly supported the renal protective potential of Smg in DKD patients.

Additionally, we tested the collected clinical serum and urine samples for indicators related to ferroptosis, and found that the change of GSH significantly increased in the DKD/Smg group while the level of Fe^2+^, MDA, and 4‐HNE decreased compared to the control group (Figure 1I–P). These markers could be used to represent the degree of renal ferroptosis in the kidneys of DKD patients.^[^ ^8^ ^]^ As expected, treatment with Smg markedly inhibited ferroptosis in both the serum and urine of DKD/Smg patients.

### 2.2 Semaglutide Treatment and Ferroptosis Inhibition Relieve Kidney Injury in Diabetic Mice

**Figure 2:**
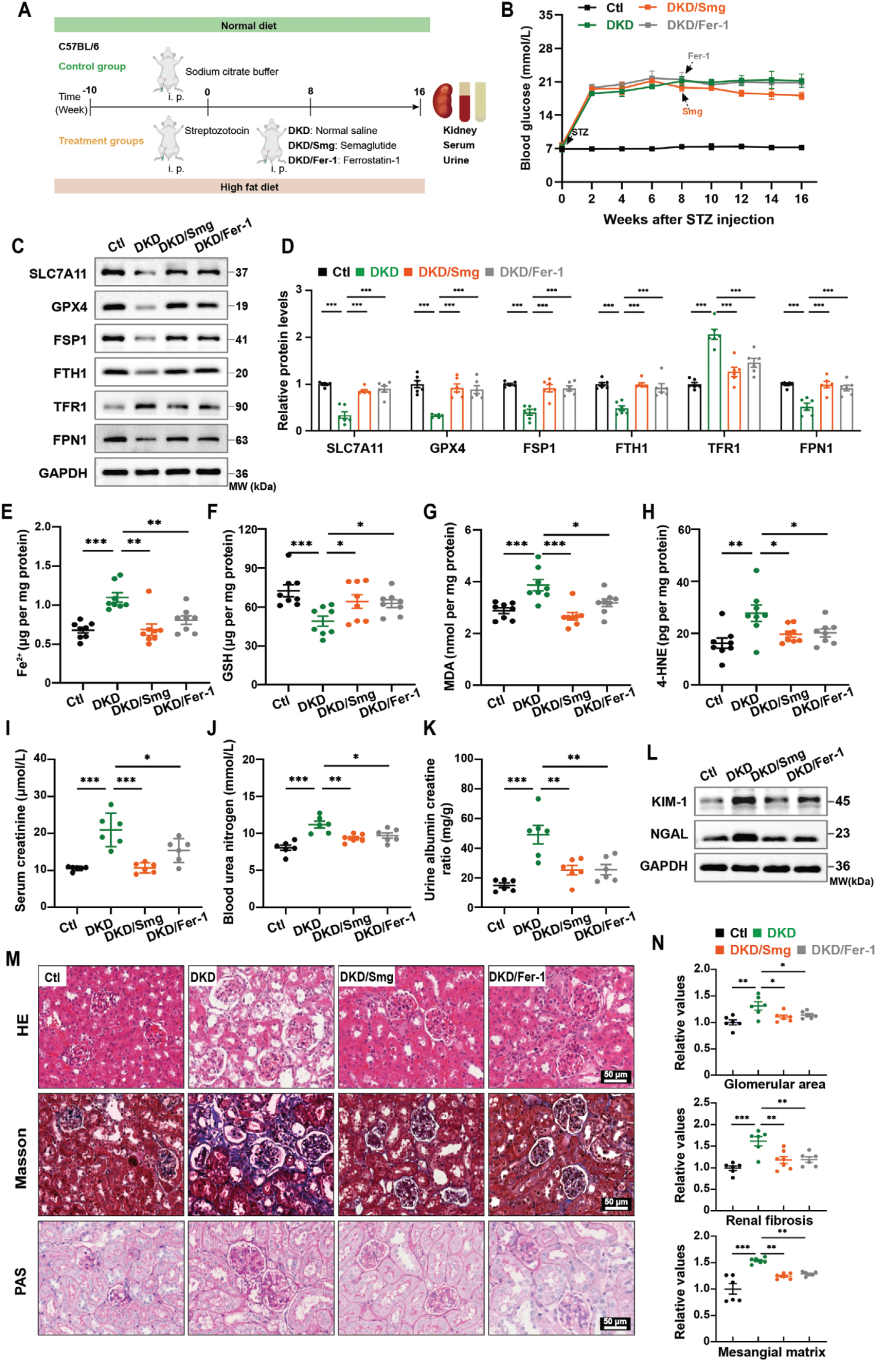
Smginhibits ferroptosis and reduces kidney injury in DKD mice. A) Schematic illustration of dosing regimen for control (Ctl), DKD, DKD/Smg, and DKD/Fer‐1 groups. B) Graphs of blood glucose changes after STZ injection (40 mg kg^−1^, five consecutive days) in four groups of mice. C, D) Immunoblot analysis and quantification of proteins associated with ferroptosis in kidney tissues of Ctl, DKD, DKD/Smg, and DKD/Fer‐1 mice. E–H) Quantification of Fe^2+^, GSH, MDA, and 4‐HNE of kidney tissues in the indicated groups. I‐K) The concentration of kidney injury biomarkers in the indicated groups. L) Immunoblot of renal tubular injury markers of kidney tissues in the indicated groups. M, N) Representative images and quantification of H&E, Masson, and PAS staining of kidney tissues in Ctl, DKD, DKD/Smg, and DKD/Fer‐1 groups. DKD mice received Smg (DKD/Smg, 60 µg kg^−1^, twice a week) or Fer‐1 (DKD/Fer‐1, 1 mg kg^−1^, daily) for eight weeks. Fer‐1: Ferrostatin‐1; SLC7A11, solute carrier family 7 member 11; GPX4, glutathione peroxidase 4; FSP1, ferroptosis suppressor protein 1; FTH1, ferritin heavy chain; TFR1, transferrin receptor 1; FPN1, ferroportin; GSH: glutathione; MDA: malondialdehyde; 4‐HNE: 4‐hydroxynonenal; KIM‐1: Kidney injury molecule 1; NGAL: Neutrophil gelatinase‐associated lipocalin. Data are presented as mean ± standard error (*n* ≥ 6). Statistical comparison was performed using one‐way ANOVA with a Tukey post‐hoc analysis, *p < 0.05, **p < 0.01, ***p < 0.001.

To investigate the effect of Smg on renal protection in vivo, we established the DKD mouse model using a high‐fat diet combined with streptozotocin (STZ) administration (**Figure** **2**A). As shown in Figure 2B, one week after 5 consecutive days of STZ injections, the blood glucose levels of mice in the DKD, Smg, and Fer‐1 (Ferrostatin‐1) groups exceeded 16.7 mmol L^−1^ and exhibited parallel trajectories in hyperglycemia progression until Smg had a slight effect on lowering blood glucose levels. In contrast, the control mice maintained blood glucose levels ≈7 mmol L^−1^.

We initially detected the ferroptosis‐related proteins to validate the impact of Smg on ferroptosis in diabetic kidney tissues. The expression of SLC7A11, GPX4, FSP1, FTH1, and FPN1 were suppressed, while TFR1 was elevated in the kidney injury induced by diabetes. However, consistent with the Fer‐1 group, diabetic mice treated with Smg showed increased levels of anti‐ferroptosis proteins (SLC7A11, GPX4, FSP1, FTH1, and FPN1) and decreased TFR1 (Figure 2C,D). Levels of Fe^2+^, MDA, and 4‐HNE in diabetic kidney tissue surpassed those in control mice, whereas Smg effectively restored these levels to those comparable to the Fer‐1 group. Moreover, the suppressed GSH level in diabetic mice was successfully restored by Smg (Figure 2E–H). In terms of renal function valuation, we found that the biochemical indicators of kidney injury, such as serum creatinine, blood urea nitrogen, urine albumin creatine ration (UACR) and urinary albumin were in a discernible elevation in diabetic mice as opposed to their control counterparts. Additionally, mice with interventions of Smg and ferroptosis inhibition showed a notable reduction in the aforementioned indicators (Figure 2I–K; Figure S2A, Supporting Information). Similarly, the levels of renal tubular injury markers *N*‐acetyl‐*β*‐glucosaminidase (NAG), transferrin, kidney injury molecule 1 (KIM‐1), and neutrophil gelatinase‐associated lipocalin (NGAL) were significantly increased in the DKD group, whereas their expression was markedly repressed under Smg treatment and ferroptosis inhibition (Figure 2L; Figure S2B–D, Supporting Information).

As illustrated by H&E and PAS staining, diabetes‐induced renal injury is characterized by marked glomerular hypertrophy, significant mesangial matrix expansion, vacuolar degeneration, and partial shedding of some renal tubular epithelial cells. The production of extracellular matrix proteins (e.g., collagen) serves as an index of fibrosis in the injured kidney.^[^ ^31^ ^]^ Masson staining primarily revealed the deposition of collagen fiber and expansion of mesangial matrix in DKD mice. However, treatment with Smg and Fer‐1, respectively, similarly attenuated these histopathologic changes compared to the DKD mice (Figure 2M,N). These results highlighted the vital role of Smg‐mediated ferroptosis inhibition in renal protection in DKD mice.

### 2.3 Semaglutide Attenuates Inflammation and Fibrosis in Diabetic Mice

**Figure 3:**
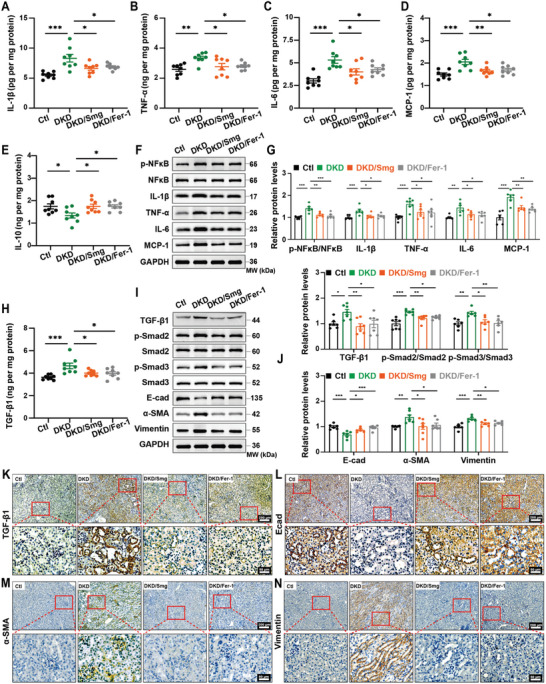
Smg attenuates kidney inflammation and fibrosis in the mice model of DKD. A–E) ELISA quantification of IL‐1β, TNF‐α, IL‐6, MCP‐1, and IL‐10 in kidney tissues from three different mice models. F, G) Immunoblot analysis and quantification of proteins associated with inflammation in kidney tissues of Ctl, DKD, DKD/Smg, and DKD/Fer‐1 mice. H) ELISA quantification of TGF‐β1. I, J) Immunoblot analysis and quantification of proteins associated with fibrosis in kidney tissues of Ctl, DKD, DKD/Smg, and DKD/Fer‐1 mice. K–N) Representative immunostaining images related to fibrosis of kidney tissues in Ctl, DKD, DKD/Smg, and DKD/Fer‐1 groups. DKD mice received Smg (DKD/Smg, 60 µg kg^−1^, twice a week) or Fer‐1 (DKD/Fer‐1, 1 mg kg^−1^, daily) for eight weeks. Fer‐1: Ferrostatin‐1; NFκB, nuclear factor kappa B; IL‐1β, interleukin‐1β; TNF‐α, tumor necrosis factor‐α; IL‐6, interleukin‐6; MCP‐1, monocyte chemoattractant protein‐1; TGF‐β1, transforming growth factor‐beta 1; E‐cad, E‐cadherin; α‐SMA, alpha‐smooth muscle action. Data are presented as mean ± standard error (*n* ≥ 6). Statistical comparison was performed using one‐way ANOVA with a Tukey post‐hoc analysis, *p < 0.05, **p < 0.01, ***p < 0.001.

The activation of NF‐κB is characterized by increased phosphorylation of its p65 subunit and elevated expression of inflammatory cytokines, including interleukin‐1*β* (IL‐1*β*), tumor necrosis factor‐*α* (TNF‐*α*), interleukin‐6 (IL‐6), and monocyte chemoattractant protein‐1 (MCP‐1).^[^ ^32^ ^]^ We found that the phosphorylation of p65 and the expression of these cytokines were significantly increased in DKD kidneys, as examined with ELISA analysis (**Figure** **3**A–D), and western blot (Figure 3F,G). Treatment with either Smg or Fer‐1 could decrease those factors in DKD kidneys. Additionally, the expression of anti‐inflammatory cytokine interleukin‐10 (IL‐10) was increased in DKD kidneys following treatment with Smg and Fer‐1 (Figure 3E). Transforming growth factor‐beta 1 (TGF‐*β*1) is a key pro‐fibrotic factor that drives collagen accumulation and fibrosis in the kidney.^[^ ^33^ ^]^ We identified an enrichment of TGF‐*β*1, phosphorylation of Smad2/3, and the dysregulation of canonical fibrotic markers, including E‐cadherin (E‐Cad), alpha‐smooth muscle actin (*α*‐SMA), and Vimentin in DKD kidneys by ELISA analysis (Figure 3H), western blot (Figure 3I,J), and immunohistochemical staining (Figure 3K–N). Upon treatment with either Smg or Fer‐1, these fibrotic indices were normalized in DKD kidneys. These results suggested that Smg could significantly mitigate renal inflammation and fibrosis in DKD mice.

### 2.4 Semaglutide Mitigates Ferroptosis in HK‐2 Cells under the HGL Condition

**Figure 4:**
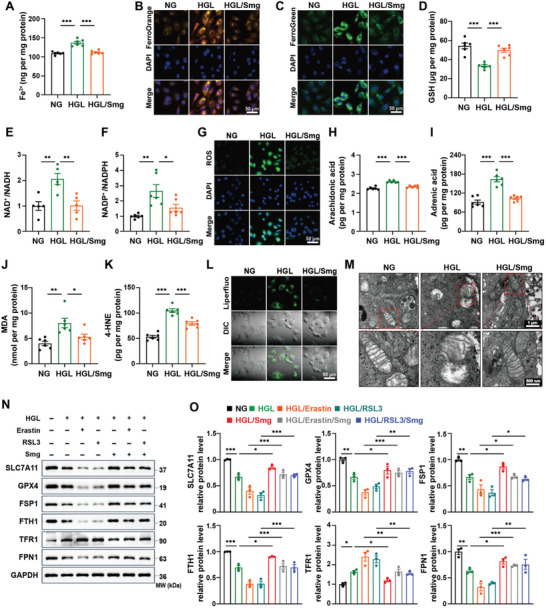
Smg alleviates ferroptosis in HK‐2 cells induced by a high concentration of glucose and lipid (i.e., HGL). A) Quantification of intracellular Fe^2+^. B, C) Confocal imaging of cytosolic and mitochondrial ferrous iron (Fe^2+^) by fluorescent FerroOrrange and FerroGreen probe, respectively. D–F). Quantification of intracellular GSH, NAD^+^/NADH, and NADP^+^/NADPH. G) Confocal imaging of intracellular ROS. H–K) Quantification of intracellular arachidonic acid, adrenic acid, MDA, and 4‐HNE. L) Confocal imaging of lipid peroxides. M) Mitochondrial morphology analysis by transmission electron microscope. N, O) Immunoblot analysis and quantification of proteins associated with ferroptosis of HK‐2 cells in the indicated groups. HK‐2 cells were cultured in NG: 5.5 mM glucose; HGL: 35 mM glucose and 120 µM palmitic acid/PA; HGL/Smg: HGL plus 400 nM Smg; ferroptosis inducers: 0.3 µM RSL3 or 4 µM Erastin. All of these assays were performed after the HK‐2 cells culturing for 48 hours under different conditions. GSH: glutathione; ROS: reactive oxygen species; MDA: malondialdehyde; 4‐HNE: 4‐hydroxynonenal; SLC7A11, solute carrier family 7 member 11; GPX4, glutathione peroxidase 4; FSP1, ferroptosis suppressor protein 1; FTH1, ferritin heavy chain; TFR1, transferrin receptor 1; FPN1, ferroportin. Data are presented as mean ± standard error (*n* ≥ 3). Statistical comparison was performed using one‐way ANOVA with a Tukey post‐hoc analysis, *p < 0.05, **p < 0.01, ***p < 0.001.

Immunofluorescence analysis revealed that the GLP‐1 receptor (GLP‐1R) was overexpressed in the renal tubular epithelial cells of the kidney tissue (Figure S2E, Supporting Information). To mimic a hyperglycemic and hyperlipidemic (HGL) condition in DKD in vitro, we determined the cellular intervention concentration by cell viability assay (Figure S3A–C, Supporting Information) and treated HK‐2 cells with a high concentration of glucose (35 mM) and palmitic acid (120 µM) resulting in an induction of a ferroptotic state. Transcriptomic analysis revealed that angiopoietin‐like 4 (ANGPTL4), a critical regulator of glucose homeostasis and lipid metabolism, was upregulated in the HGL group (Figure S3D, Supporting Information).^[^ ^34^ ^]^ ANGPTL4 has been reported to activate reduced nicotinamide adenine dinucleotide phosphate (NADPH) oxidase 2, leading to oxidative stress and ferroptosis.^[^ ^35^, ^36^ ^]^ We also found that cytosolic and mitochondrial ferrous ion (Fe^2+^) was elevated in the HGL group, which could be reversed under Smg treatment (**Figure** **4**A–C; Figure S3E,F, Supporting Information). Fe^2+^ is well‐known for producing hydroxyl radicals via the Fenton reaction, resulting in the augmentation of reactive oxygen species (ROS) and lipid peroxides.^[^ ^37^ ^]^ Moreover, HGL treatment caused a diminishment of the predominant intracellular antioxidant, GSH and NAD(P)H, and induced an elevation of ROS (Figure 4D–G; Figure S3G, Supporting Information), whereas Smg reversed the above changes. NAD(P)H acts as a critical electron donor and plays an essential role in maintaining redox homeostasis by facilitating the generation of reduced forms of antioxidants,^[^ ^38^ ^]^ which has also been identified as a biomarker for the sensitivity of ferroptosis.^[^ ^39^, ^40^ ^]^ These results suggested that treatment with Smg counteracts the HGL‐induced iron metabolism disorder and redox impairment.

Additionally, as evidenced by the extensive lipid peroxidation and the increase of arachidonic acid (AA), adrenic acid (AdA), malondialdehyde (MDA), and 4‐hydroxynonenal (4‐HNE), the degradation end‐products of lipid peroxides and key ferroptosis biomarkers (Figure 4H–L; Figure S3H, Supporting Information) upon HGL treatment.^[^ ^41^ ^]^ AA and AdA are the critical polyunsaturated fatty acids essential for the synthesis of ferroptotic phospholipid hydroperoxide (PE‐AA‐OOH/PE‐AdA‐OOH).^[^ ^42^, ^43^, ^44^ ^]^ As the key ferroptosis biomarkers, MDA and 4‐HNE are the degradation end‐products of lipid peroxides.^[^ ^45^ ^]^ Lipid peroxidation occurs in plasma and organelle membranes, including mitochondria.^[^ ^46^ ^]^ Consequently, ferroptotic cells are often associated with changes in mitochondria morphology and function.^[^ ^47^, ^48^, ^49^ ^]^ Altered mitochondrial morphology is an important hallmark of ferroptosis, as visualized through mitochondrial transmission electron microscopy, we observed that, in comparison to the HGL group, condensed membrane densities and reduced mitochondrial ridges were significantly alleviated under the influence of Smg intervention (Figure 4M).

We further determined the cellular intervention concentration by cell viability assay (Figure S3I–L, Supporting Information) and detected ferroptosis‐related proteins by western blot, and found that the HGL challenge decreased the expression of SLC7A11, GPX4, and FSP1, three key regulators of ferroptosis,^[^ ^50^ ^]^ whereas their expression could be restored by Smg (Figure 4N,O). In addition, HGL treatment resulted in an elevation of transferrin receptor 1 (TFR1) and a decrease of ferroportin (FPN1) and ferritin (FTH1) in HK‐2 cells, suggesting a change in iron metabolism. Transferrin shows high affinity with ferric iron, and the Fe^3+^‐bearing transferrin docks with the membrane‐bound TFR1 before internalization via the clathrin‐mediated endocytosis, which is a critical route of cellular iron intake.^[^ ^51^ ^]^ FPN1 is responsible for the export of labile iron out of cells.^[^ ^52^ ^]^ As expected, we found that HGL treatment induced the downregulation of FPN1, contributing to the accumulation of Fe^2+^ in the labile iron pool (Figure 4N,O). Moreover, ferritin is an iron‐storage protein, and its downregulation under a diabetic milieu favors the enrichment of intracellular Fe^2+^ and hence ferroptotic cell death. Notably, the expression of these iron metabolism regulatory proteins was restored with Smg treatment. Furthermore, ferroptosis was evident in HK‐2 cells treated with canonical ferroptosis inducers (Erastin or RSL3) based on HGL condition; however, Smg was able to rescue the viability of HK‐2 cells under these challenges (Figure 4N,O), highlighting its potential for inhibiting ferroptosis in HK‐2 cells exposed to HGL condition.

### 2.5 Semaglutide Inhibits Ferroptosis and Alleviates Inflammation, Fibrosis, and Kidney Damage in HK‐2 and Primary Renal Tubular Cells under HGL Conditions

**Figure 5:**
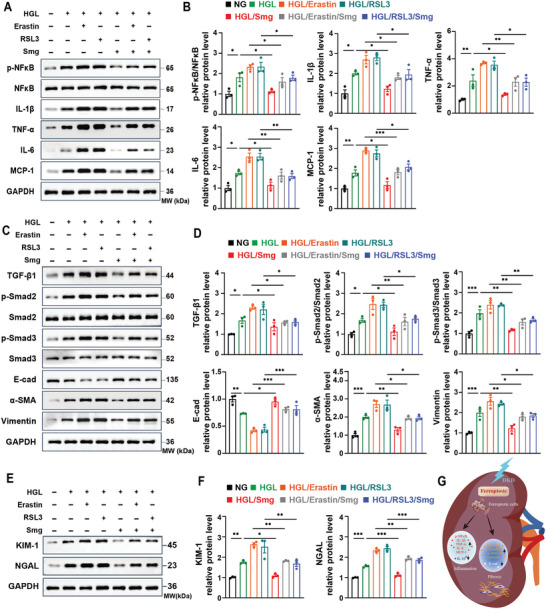
Smg alleviates inflammation, fibrosis, and kidney damage via ferroptosis inhibition in HK‐2 cells. A, B) Immunoblot analysis and quantification of proteins associated with inflammation of HK‐2 cells in the indicated groups. C, D) Immunoblot analysis and quantification of proteins associated with fibrosis of HK‐2 cells in the indicated groups. E, F) Immunoblot analysis and quantification of proteins associated with tubular damage of HK‐2 cells in the indicated groups. G) Schematic illustration of diabetes‐induced renal ferroptosis promoting renal inflammation and fibrosis. HK‐2 cells were cultured in NG: 5.5 mM glucose; HGL: 35 mM glucose and 120 µM palmitic acid/PA; HGL/Smg: HGL plus 400 nM Smg; ferroptosis inducers: 0.3 µM RSL3 or 4 µM Erastin. All of these assays were performed after the HK‐2 cells culturing for 48 hours under different conditions. NFκB, nuclear factor kappa B; IL‐1β, interleukin‐1β; TNF‐α, tumor necrosis factor‐α; IL‐6, interleukin‐6; MCP‐1, monocyte chemoattractant protein‐1; TGF‐β1, transforming growth factor‐beta 1; E‐cad, E‐cadherin; α‐SMA, alpha‐smooth muscle action; KIM‐1: Kidney injury molecule 1; NGAL: Neutrophil gelatinase‐associated lipocalin. Data are presented as mean ± standard error (*n* ≥ 3). Statistical comparison was performed using one‐way ANOVA with a Tukey post‐hoc analysis, * *p* < 0.05, **p < 0.01, ***p < 0.001.

Oxidative stress often leads to the activation of the transcription factor, NF‐κB, followed by inflammatory responses.^[^ ^53^ ^]^ We found that HGL treatment increased the phosphorylation of the p65 subunit of NF‐κB and elevated expression of pro‐inflammatory cytokines, including IL‐1β, TNF‐α, IL‐6, and MCP‐1, whereas Smg could normalize the levels of these factors (**Figure** **5**A,B). Diabetic kidney injury usually results in renal fibrosis, which is regulated by TGF‐*β*1.^[^ ^33^ ^]^ Our results showed that HGL treatment increased the expression of TGF‐*β*1 and the phosphorylation of Smad2 and Smad3 as well as the expression of *α*‐SMA and Vimentin, but decreased the expression of E‐Cad in HK‐2 cells. However, treatment with Smg normalized these levels (Figure 5C,D). In addition, we observed that ferroptosis further exacerbated inflammation and fibrosis in HK‐2 cells treated with Erastin or RSL3 under HGL conditions, while Smg mitigated these changes (Figure 5A–D). These results supported the role of Smg‐mediated ferroptosis inhibition in attenuating inflammation and fibrosis under HGL conditions. We also analyzed kidney injury biomarkers and found that the levels of both KIM‐1 and NGAL were significantly increased under the HGL condition. Furthermore, their levels were exacerbated by the addition of Erastin or RSL3. In contrast, Smg treatment markedly repressed these biomarkers through ferroptosis inhibition, indicating Smg could protect against renal injury by inhibiting ferroptosis under HGL conditions (Figure 5E,F).

Additionally, we utilized the primary renal tubular cells to analyze the rescue effect of Smg under HGL conditions and ferroptosis stress (Figure S4, Supporting Information). Cell exposure to HGL conditions significantly reduced the level of GPX4 and SLC7A11, accompanied by increased TFR1, indicating the presence of ferroptosis under HGL conditions. Moreover, the concurrent exposure of primary renal tubular cells to HGL and RSL3 led to an upregulation of inflammatory markers such as IL‐1β and IL‐6, as well as fibrosis markers including TGF‐β1 and vimentin, while the expression of E‐cad was suppressed. Notably, RSL3‐induced ferroptosis also enhanced the expression of KIM‐1. However, the administration of Smg potently reversed the changes of these markers, demonstrating that Smg can protect primary renal tubular cells against the detrimental effects under HGL and ferroptosis stress, thereby mitigating inflammation, fibrosis, and renal injury.

### 2.6 Semaglutide Upregulates KLB via GLP‐1R/cAMP/PKA/CREB Pathway

**Figure 6:**
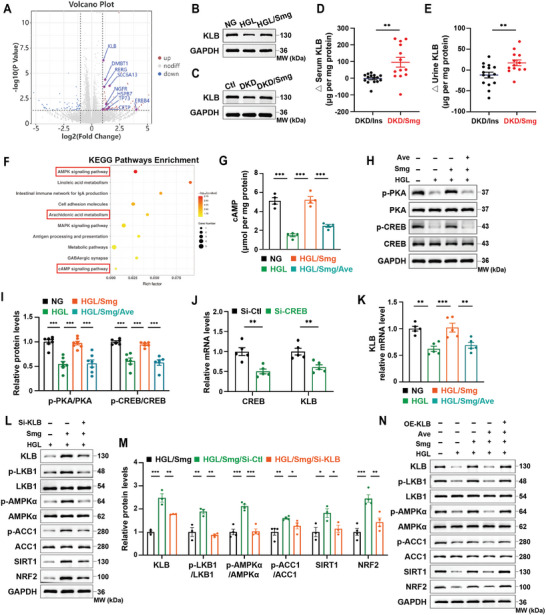
Smg regulates the AMPK signaling pathway via KLB under HGL condition. A) Volcano plot showing the upregulated genes in Smg‐incubated HK‐2 cells under HGL condition. B, C) Immunoblot analysis of KLB protein expression in vitro and vivo experiments under diabetic condition and combined with Smg treatment. D, E) The changes of serum and urine concentration of soluble β‐Kloth in DKD/Ins and DKD/Smg patients. F) Bubble diagram of KEGG pathways enrichment analysis for the top ten pathways under Smg treatment. G) The intracellular concentration of cAMP in HK‐2 cells treated by NG, HGL, HGL/Smg, HGL/Smg/Ave. H, I) Immunoblot analysis and quantification of proteins associated with the downstream of GLP‐1R activation in the indicated groups. J) mRNA quantification of KLB and CREB in HK‐2 cells post CREB knockdown by small interfering RNA (siCREB, 100 nM). K) mRNA quantification of KLB in HK‐2 cells post‐treatment by HGL, HGL/Smg, and HGL/Smg/Ave. L, M) Immunoblot analysis and quantification of KLB and proteins associated with the AMPK pathway in HGL, HGL/Smg, and HGL/Smg/Si‐KLB groups. N) Immunoblot analysis of KLB and proteins associated with the AMPK pathway in NG, HGL, HGL/Smg, HGL/Smg/Ave and HGL/Smg/Ave/OE‐KLB groups. HK‐2 cells were cultured in NG: 5.5 mM glucose; HGL: 35 mM glucose and 120 µM palmitic acid/PA; HGL/Smg: HGL plus 400 nM Smg; HGL/Smg/Ave: HGL plus Smg and 300 nM Avexitide/Ave; Si‐KLB: 100 nM si‐RNA of KLB; OE‐KLB: 1.0 µg mL^−1^ plasmid DNA of KLB. All of these assays were performed after the HK‐2 cells culturing for 48 hours under different conditions. KLB, β‐Klotho; cAMP: cyclic adenosine monophosphate; Ave: Avexitide, a glucagon‐like peptide‐1 (GLP‐1) receptor inhibitor; PKA: Protein kinase A; CREB: cAMP‐response element binding protein; LKB1, liver kinase beta 1; AMPK, AMP‐activated protein kinase; ACC, acetyl‐CoA carboxylase; SIRT1, sirtuin 1; NRF2, nuclear factor erythroid 2‐related factor 2. Data are presented as mean ± standard error (*n* ≥ 3). Statistical comparison was performed using unpaired two‐tailed Student's *t*‐test (J) or one‐way ANOVA coupled with a Tukey post‐hoc analysis, *p < 0.05, **p < 0.01, ***p < 0.001.

To investigate how Smg inhibited ferroptosis, we performed transcriptome sequencing in HGL‐treated HK‐2 cells with or without co‐treatment of Smg. Our analysis revealed that treatment with Smg significantly increased the expression of multiple genes in HK‐2 cells (**Figure** **6**A). Among those genes, KLB attracted our attention, which may play a role in Smg‐mediated inhibition of ferroptosis. We confirmed the suppression of KLB in HGL‐treated HK‐2 cells and the kidney tissue of DKD mice, while Smg notably restored KLB levels, as evidenced by western blot analysis (Figure 6B,C; Figure S5A,B, Supporting Information). Additionally, we observed that treatment with Smg significantly elevated the levels of soluble KLB in the blood and urine of DKD patients treated with Smg compared to those treated with insulin (DKD/Ins) (Figure 6D,E). Collectively, these findings suggested that the Smg‐mediated increase of KLB both in vitro and in vivo may mitigate diabetic kidney injury.

From the KEGG pathways enrichment result, the arachidonic acid metabolism pathway that plays an important role in lipoperoxidation was enriched, providing strong support for the effect of Smg on ferroptosis (Figure 6F). The KEGG results also showed an enrichment of the cyclic adenosine monophosphate (cAMP) signaling pathway, while GLP‐1RAs can activate the cAMP signaling pathway to regulate the transcription of various genes.^[^ ^54^ ^]^ Hence, we postulated that the binding of Smg to GLP‐1R could activate cAMP‐PKA (protein kinase A)‐CREB (cAMP‐response element‐binding protein) signaling pathway.^[^ ^55^ ^]^ We found that the concentration of cAMP was significantly reduced in HK‐2 cells post HGL treatment, but it was restored upon treatment with Smg. However, co‐treatment with a GLP‐1R antagonist (avexitide/Ave) markedly mitigated the cAMP level in these cells (Figure 6G; Figure S5C, Supporting Information). Furthermore, treatment with Smg increased the phosphorylation of PKA and CREB, whereas treatment with HGL and Ave hindered their activation (Figure 6H,I). Consequently, the knockdown of CREB and intervention with Ave both suppressed the mRNA expression of KLB (Figure 6J,K).

### 2.7 Semaglutide Regulates the AMPK Signaling Pathway via KLB under HGL Condition

KEGG pathways enrichment result concluded that the genes mainly affected by Smg were enriched in the AMP‐activated protein kinase (AMPK) signaling pathway (Figure 6F). It has been reported that α‐klotho (KLA), another klotho protein, could protect against diabetic kidney disease by promoting the phosphorylation of AMPK, a central regulator of energy homeostasis.^[^ ^56^ ^]^ Our study observed a diminishment of KLA in DKD patients, DKD mice, and HGL‐cultured HK‐2 cells, which can be restored by Smg (Figure S5D–G, Supporting Information). We therefore hypothesized that Smg affects the KLB/AMPK signaling pathway to inhibit ferroptosis. The schematic mechanism illustration of how Smg inhibits ferroptosis is shown in Figure S9 (Supporting Information). We verified that Smg regulates the AMPK signaling pathway through KLB by KLB knockdown experiment under the HGL/Smg condition (Figure 6L,M). AMPK activation is often induced through the phosphorylation of liver kinase beta 1 (LKB1).^[^ ^57^ ^]^ Our findings showed that Smg promotes the expression of KLB, which further ameliorates HGL‐induced injury via the LKB1/AMPK axis (Figure 6L,M). Evidence showed that AMPK activation regulates lipid metabolism, resulting in ferroptosis inhibition.^[^ ^58^ ^]^ AMPK activation could phosphorylate the acetyl‐CoA carboxylase (ACC) that catalyzed the conversion of acetyl‐CoA to malonyl‐CoA. Malnoyl‐CoA is critical for the synthesis of AA and AdA. The esterification of AA/AdA with phosphoethanolamine (PE) generates tailored lipids (PE‐AA/PE‐AdA) that induce ferroptosis post peroxidation.^[^ ^59^ ^]^ Therefore, our results suggested that Smg indirectly inhibited ferroptosis by mediating the synthesis of ferroptotic lipids via the AMPK/ACC pathway.

**Figure 7:**
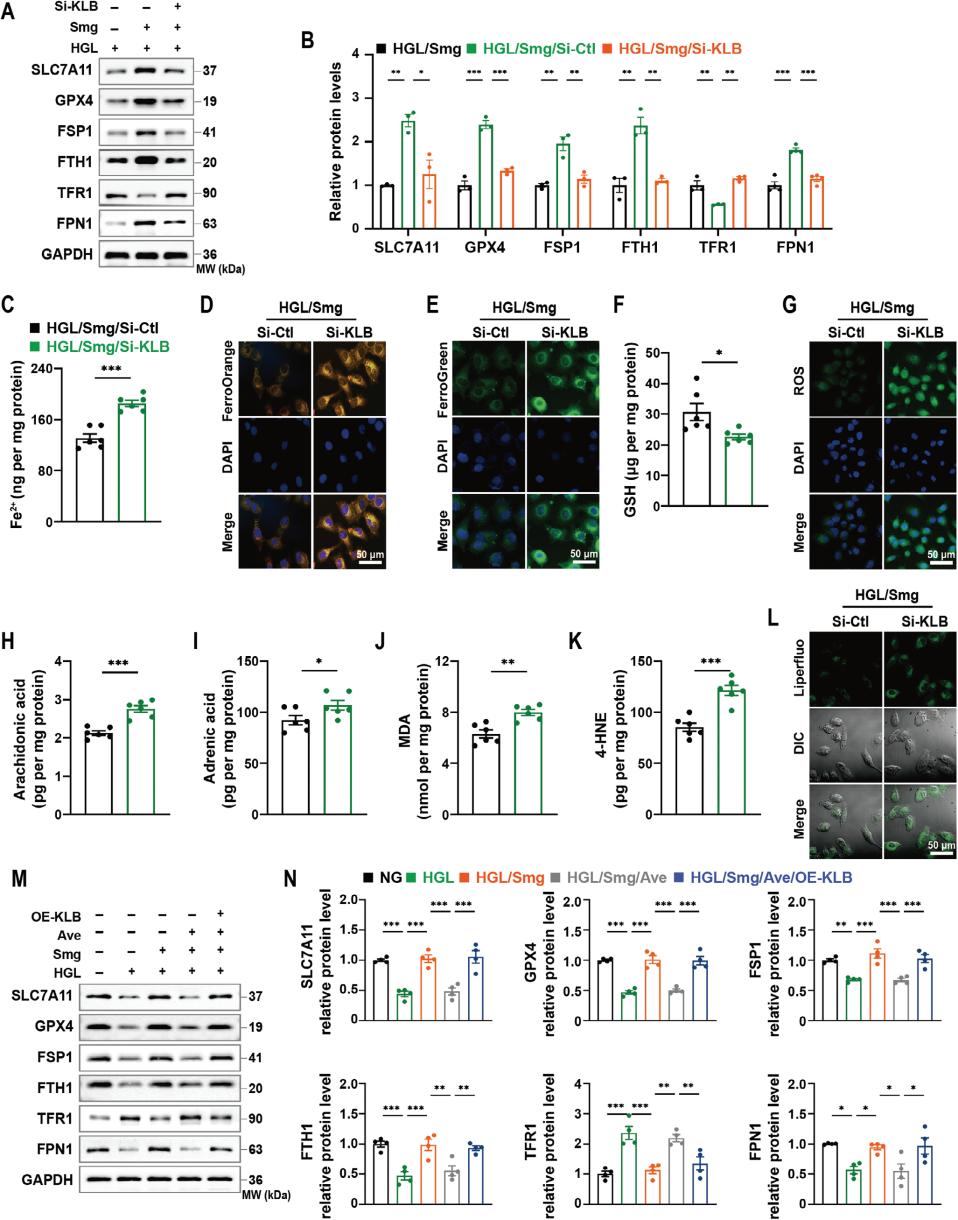
KLB silence counteracts Smg‐induced ferroptosis inhibition in HGL‐treated HK‐2 cells. A, B) Immunoblot analysis and quantification of proteins associated with ferroptosis in HK‐2 cells treated by HGL, HGL/Smg, HGL/Smg/Si‐KLB. C) Quantification of intracellular Fe^2+^ in HK‐2 cells treated by Si‐control or Si‐KLB under the HGL/Smg condition. D, E) Confocal imaging of cytosolic and mitochondrial ferrous iron (Fe^2+^) by fluorescent FerroOrrange and FerroGreen probe, respectively. F) Quantification of intracellular GSH. G) Confocal imaging of intracellular ROS. H–K) Quantification of intracellular arachidonic acid, adrenic acid, MDA, and 4‐HNE. L) Confocal imaging of lipid peroxides. M, N) Immunoblot analysis and quantification of proteins associated with ferroptosis in HK‐2 cells treated by NG, HGL, or HGL/Smg, HGL/Smg/Ave, HGL/Smg/Ave/OE‐KLB. HK‐2 cells were cultured in NG: 5.5 mM glucose; HGL: 35 mM glucose and 120 µM palmitic acid/PA; HGL/Smg: HGL plus 400 nM Smg; HGL/Smg/Ave: HGL plus Smg and 300 nM Avexitide/Ave; Si‐KLB: 100 nM si‐RNA of KLB; OE‐KLB: 1.0 µg mL^−1^ plasmid DNA of KLB. All of these assays were performed after the HK‐2 cells culturing for 48 h under different conditions. GSH: glutathione; ROS: reactive oxygen species; MDA: malondialdehyde; 4‐HNE: 4‐hydroxynonenal; SLC7A11, solute carrier family 7 member 11; GPX4, glutathione peroxidase 4; FSP1, ferroptosis suppressor protein 1; FTH1, ferritin heavy chain; TFR1, transferrin receptor 1; FPN1, ferroportin. Data are presented as mean ± standard error (*n* ≥ 3). Statistical comparison was performed using the unpaired, two‐tailed Student's *t*‐test (C, F, H–K) or one‐way ANOVA with a Tukey post‐hoc analysis (B, N), *p < 0.05, **p < 0.01, ***p < 0.001.

SIRT1 is an NAD^+^‐dependent deacetylase that mediates the metabolic response to nutrient availability.^[^ ^60^ ^]^ It modulates the activity of a myriad of downstream targets, such as the nuclear factor erythroid 2‐related factor 2 (NRF2).^[^ ^61^ ^]^ NRF2 is the leading regulator of the antioxidant response and governs the transcription of multiple ferroptosis‐related genes (e.g., SLC7A11, GPX4, and FSP1).^[^ ^62^ ^]^ We found that treatment with Smg restored the expression of SIRT1 and NRF2 as well as the phosphorylation of AMPK (Figure 6L,M), and increased the expression of FTH1 and FPN1, but decreased the expression of TFR1 (**Figure** **7**A), resulting in a reduction of Fe^2+^ in the labile iron pool and a suppression of ferroptosis (Figure 4A–C). Thus, the normalization of iron metabolism and the suppression of ferroptosis by Smg in HGL‐treated HK‐2 cells might be mediated by the AMPK/SIRT1/NRF2 pathway (Figure 6L,M). Furthermore, we found that the silence of KLB blocked the effect of Smg on the phosphorylation of LKB1 and AMPK as well as the expression of SIRT1, and NRF2 under HGL/Smg condition (Figure 6L,M). These results suggested that the silence of KLB affected the regulation of Smg on the AMPK signaling pathway under the HGL condition.

To further illustrate the role of KLB in the regulation of the AMPK signaling pathway under HGL condition, we performed KLB overexpression in HGL‐intervened cells, and found that KLB overexpression was followed by activation of the AMPK signaling pathway and elevated SIRT1 and NRF2 levels (Figure S5H, I, Supporting Information).

The downregulation of KLB induced by avexitide (Ave) resulted in the suppression of the AMPK/ACC/SIRT1/NRF2 pathways. We subsequently performed rescue experiments with KLB overexpression following Ave intervention and found that Smg regulated the AMPK/ACC/SIRT1/NRF2 signaling pathway via KLB was dependent on the GLP‐1R pathway (Figure 6N; Figure S5J, Supporting Information).

Proliferator‐activated receptor gamma coactivator‐1α (PGC‐1α), a master regulator of mitochondrial biogenesis and cellular energy metabolism,^[^ ^63^ ^]^ is essential for maintaining kidney function but is usually depleted in diabetic mice.^[^ ^64^ ^]^ Intriguingly, treatment with Smg strikingly restored the level of PGC‐1α in injured DKD kidneys and HK‐2 cells, possibly via the KLB/AMPK/SIRT1 pathway (Figure S5K–N, Supporting Information).^[^ ^65^ ^]^ These results highlighted the potency of Smg in protecting renal function from diabetic kidney injury.

### 2.8 Knockdown of KLB Counteracts the Action of Semaglutide on Ferroptosis

Compared with the HGL group, Smg upregulated the expression of ferroptosis regulatory proteins SLC7A11, GPX4, FSP1, FTH1, and FPN1, but decreased TFR1, suggesting that Smg inhibited ferroptosis under HGL condition. When further carried out the knockdown of KLB with small interfering RNAs (siRNAs), the inhibitory effect of Smg on ferroptosis was counteracted (**Figure** **7**A,B), suggesting that the silence of KLB boosts ferroptosis in Smg‐treated HK‐2 cells under HGL condition.

To determine the role of KLB in regulating biochemical indicators of ferroptosis, we knocked down KLB in cells treated with HGL combined with Smg. We found that silence of KLB significantly increased the levels of ferroptosis markers, including Fe^2+^, AA, AdA, MDA, and 4‐HNE, but decreased the level of GSH (Figure 7C,F,H–K). Silence of KLB also increased cytosolic and mitochondrial Fe^2+^, accompanied by an increase of ROS and lipid peroxides in HGL/Smg HK‐2 cells (Figure 7D,E,G,L; Figure S6A, Supporting Information). We also determined whether overexpression of KLB could rescue the effect of GLP‐1R inhibition on the key ferroptosis regulators. We found that treatment with Ave modulated the expression of SLC7A11, GPX4, FSP1, FTH1, TFR1, and FPN1 in HK‐2 cells under HGL/Smg condition, resulting in enhanced ferroptosis (Figure 7M,N). In contrast, treatment with the KLB polyplex (OE‐KLB) restored the level of SLC7A11, GPX4, and FSP1; normalized iron metabolism, as evidenced by an increase of the expression of FTH1 and FPN1 and a decrease of TFR1 (Figure 7M,N).

Moreover, we revealed that the expression of KLB and the activity of the AMPK were diminished in primary renal tubular cells under the HGL conditions. At the same time, Smg treatment enhanced the KLB expression and stimulated the AMPK signaling pathway. Conversely, we noted that KLB knockdown decreased the KLB level, followed by a subsequent suppression of the AMPK signaling pathway (Figure S6B, C, Supporting Information). Additionally, the silencing of KLB led to a significant increase in ferroptosis markers such as TFR1, MDA, and 4‐HNE and a decrease in the levels of SLC7A11, GPX4, FSP1, FTH1, FPN1, and GSH in primary renal tubular cells (Figure S6D–H, Supporting Information), indicating Smg could regulate the AMPK signaling pathway and ferroptosis via the action of KLB.

### 2.9 Treatment with other Types of GLP‐1RAs also Inhibits Ferroptosis

HK‐2 cells were also treated with either liraglutide (Lrg) or dulaglutide (Dlg) under HGL condition to determine whether other types of GLP‐1RAs show similar effect on the inhibition of ferroptosis as Smg. As expected, we found that treatment with Lrg and Dlg increased the viability of HK‐2 cells under a diabetic milieu (Figure S7A,B, Supporting Information). Additionally, treatment with Lrg and Dlg significantly increased the expression of KLB mRNA and protein in HGL‐challenged HK‐2 cells (Figure S7C–H, Supporting Information). These results suggested that Smg‐mediated ferroptosis inhibition might also be true for other types of GLP‐1RAs, and KLB could be a common target of GLP‐1RAs in the suppression of ferroptosis.

### 2.10 Semaglutide and KLB Overexpression Regulate the AMPK Signaling Pathway and Ferroptosis in Mice with Diabetic Kidney Injury

As previously described, we observed that Smg increased KLB expression and inhibited ferroptosis in the renal tissues of DKD mice (Figures 2C and 6C). We further found that the downstream factors of KLB, including the phosphorylation of LKB1, AMPK, and ACC1, as well as the expression of SIRT1, and NRF2 were dysregulated in DKD kidneys. However, treatment with Smg normalized the phosphorylation and expression of these factors in DKD kidneys, as examined by western blot analysis (Figure S8, Supporting Information).

To verify the effect of KLB on the AMPK signaling pathway and ferroptosis in vivo, we injected AVV‐Klb to overexpress Klb in DKD mice. As shown in Figure S8C–F (Supporting Information), the overexpression of Klb not only elevated the level of KLB in both NC and DKD mice, but also significantly increased the phosphorylation of LKB1, AMPK, and ACC1, along with the expression of SIRT1, NRF2, SLC7A11, GPX4, and FSP1. In addition, Klb overexpression normalized iron metabolism, as seen by an increase in the expression of FTH1, FPN1, and a decrease of TFR1. These results highlighted the vital role of the Smg‐mediated KLB/AMPK signaling pathway in ferroptosis inhibition of diabetic kidneys, which was consistent with cellular experiments.

## 3 Discussion

In this study, we demonstrated that Smg could alleviate diabetic kidney injury by inhibiting ferroptosis in DKD patients, mice, HK‐2, and primary renal tubular cells. Notably, we demonstrated the suppression of ferroptosis as well as improvement in renal function in the diabetic mice in response to the Smg used at a dose of 60 µg kg^−1^ that did not reduce blood glucose, suggesting that the renal benefit of Smg in diabetic kidney disease may be achievable independently of glucose lowering. The same result was also reported in the study of Sourris et al.^[^ ^66^ ^]^ They characterized changes in the kidney in response to GLP‐1RA (liraglutide, 50 µg kg^−1^, once daily for 20 weeks) at a dose that did not reduce blood glucose and identified that liraglutide could confer renoprotection independently of blood glucose control in an experimental model of insulin‐deficient diabetes.

Mounting research has revealed the essential role of ferroptosis in the development of diabetic nephropathy.^[^ ^8^, ^19^, ^20^ ^]^ Ferroptotic cell death is critically involved in the inflammatory response, and targeting ferroptosis could attenuate renal inflammation and fibrosis.^[^ ^67^ ^]^ In this study, treatment with Smg significantly improved renal function and suppressed ferroptosis in DKD patients, and decreased renal inflammation and fibrosis in diabetic mouse models, HK‐2, and primary renal tubular cells. To investigate the specific mechanism underlying the renal benefits of Smg, we conducted cellular transcriptomic analyses and found that KLB was up‐regulated upon Smg treatment through the activation of the cAMP/PKA/CREB pathway after binding to GLP‐1R. We also established that KLB can rescue HK‐2 cells from ferroptosis by enhancing antioxidant enzyme expression, altering iron metabolism, and lipid remodeling. Based on the cellular transcriptomics results, we revealed that the KLB‐mediated ferroptosis inhibition under Smg treatment occurs via activation of the AMPK signaling pathway. In particular, the AMPK/ACC and AMPK/NRF2 axes primarily regulated iron metabolism, fatty acid synthesis, and lipid peroxidation. In addition, proper mitochondrial biogenesis is critical for preserving kidney function.^[^ ^68^ ^]^ Our data suggested that the KLB‐activated AMPK/SIRT1 pathway can increase PGC‐1α to promote mitochondrial biogenesis. However, the silence of KLB impaired the impact of Smg on ferroptosis inhibition.

Chronic renal hypoxia induces and exacerbates oxidative stress, serving as a persistent and detrimental factor that contributes to diabetic kidney injury.^[^ ^69^ ^]^ Renal lipid deposition and reduced renal perfusion negatively affect renal oxygenation levels, leading to a hypoxic state that further aggravates kidney injury. GLP‐1RAs have been reported to increase renal medullary and cortical perfusion and maintain renal oxygenation during sodium chloride loading in healthy individuals.^[^ ^70^ ^]^ In our study, Smg effectively reduced perirenal lipid deposition, increased renal cortical perfusion, and improved renal oxygenation as evidenced by fMRI examinations. Reduced cortical oxygenation is associated with renal fibrosis, indirectly suggesting that Smg may ameliorate renal pathological changes. Inversely, it has also been demonstrated that short‐term liraglutide has no effect on RBF and does not alter local renal oxygenation.^[^ ^71^ ^]^ This discrepancy may be attributed to differences in trial design and the distinct characteristics and mechanisms of action of the drugs.

Ferroptosis in diabetic kidney injury is strongly associated with inflammation and fibrosis. Previous studies have confirmed the close association between ferroptosis and various inflammation‐related signaling pathways such as cGAS‐STING, JAK‐STAT, NLRP3, NF‐κB, and MAPK, suggesting that ferroptosis induces the release of high levels of pro‐inflammatory cytokines contributing to cellular injury.^[^ ^23^, ^24^, ^72^ ^]^ Additionally, factors such as iron overload, GPX4 inhibition, and lipid peroxidation disrupt parenchymal cells, leading to an abnormal accumulation of fibrotic lesions in tissues and promoting fibrosis.^[^ ^25^, ^27^ ^]^ Wang et al. reported that the 5/6 nephrectomy‐induced CKD and fibrosis could be interfered by modulating ferroptosis.^[^ ^73^ ^]^ In db/db mice, the ferroptosis inhibitor Fer‐1 improved renal fibrosis by inhibiting HIF‐1α/HO‐1.^[^ ^74^ ^]^ Ide et al. found that iron overload‐induced ferroptosis in severely injured proximal renal tubular cells can lead to a massive accumulation of inflammatory cells, further enhancing inflammation and fibrosis.^[^ ^75^ ^]^ Our study similarly observed that ferroptosis activates NF‐κB and TGFβ1/Smad2/3 signaling pathways in HK‐2 cells, promoting extracellular matrix deposition and fibrosis. Taken together, these findings suggest that ferroptosis is closely related to the onset and progression of inflammation and fibrosis in DKD, and may be a target for renal fibrosis treatment.

Regarding the effect of GLP‐1RAs on renal inflammation and fibrosis by inhibiting ferroptosis, previous studies have reported that GLP‐1RA liraglutide attenuated systemic inflammation^[^ ^76^ ^]^ and renal fibrosis in DKD.^[^ ^77^ ^]^ In the present study, we observed activation of the NFκB and TGF‐β1/Smads signaling pathways, along with elevated expression levels of pro‐inflammatory factors (IL‐1β, TNF‐α, IL‐6, and MCP‐1) and fibrotic proteins (α‐SMA, Vimentin), while Ecad was reduced. Following further intervention with semaglutide in response to ferroptosis inducer treatments, it was observed that ferroptosis in HK‐2 cells was inhibited followed by the reduction of inflammation and fibrosis as well as an amelioration of renal tubular injury, suggesting that semaglutide could inhibit the ferroptosis‐induced inflammation and fibrosis.

KLB is an essential component of FGFR complexes and is critical for the regulatory effects of endocrine FGF19/21 on energy expenditure, glucose, lipid metabolism, and bile acid biosynthesis, and the loss of KLB eliminates all effects of FGF21.^[^ ^19^ ^]^ Previous research has shown that the FGF21‐KLB axis can promote β cell survival and insulin biosynthesis, and this function of KLB occurs independently of FGF21.^[^ ^78^ ^]^ Meanwhile, soluble KLB is produced from the extracellular domain of the full‐length transmembrane KLB via proteolytical cleavage, acting as a hormone. There is a positive correlation between soluble klotho and transmembrane klotho.^[^ ^79^ ^]^ Akin to KLB, we found the reduced levels of KLA in DKD patients, DKD mice, and HGL‐cultured HK‐2 cells were restored by Smg. In contrast to KLB which exists in the liver, pancreas, and adipose tissues, KLA is mainly present in the kidney and functions as the FGF23 co‐receptor.^[^ ^80^ ^]^ KLA determines the regulatory effects of FGF23 on glucose metabolism and energy expenditure, respectively, playing a pivotal role in maintaining kidney health and function as an anti‐aging protein.^[^ ^81^ ^]^ DKD is often characterized by a reduction of nephron number and an increase of FGF23, a potent negative regulator of KLA, leading to decreased serum soluble KLA in patients with chronic kidney disease.^[^ ^82^, ^83^ ^]^ The protective role of KLA against diabetic nephropathy has been well documented.^[^ ^84^ ^]^ First, the renal protection of KLA is believed to involve the activation of AMPK and PGC‐1α.^[^ ^56^, ^57^ ^]^ Second, soluble KLA can directly bind to the type II TGF‐β receptor to inhibit TGF‐β signaling and renal fibrosis.^[^ ^85^ ^]^ Thirdly, KLA can suppress the transient receptor potential channel C6‐mediated calcium influx in podocytes and ameliorate albuminuria.^[^ ^86^ ^]^ Ferroptotic cell death occurs through an osmotic mechanism highlighted by calcium influx and cell swelling,^[^ ^87^ ^]^ suggesting that KLA may suppress ferroptosis by regulating osmotic pressure. Fourthly, KLA can also repress the WNT‐β‐catenin signaling involved in DKD‐associated epithelial‐mesenchymal transition, collagen accumulation, kidney fibrosis, and vascular calcification.^[^ ^88^, ^89^, ^90^ ^]^ The potency of KLA in ferroptosis inhibition has been reported in cognitive deficit models.^[^ ^91^ ^]^ Although the transcriptomic analysis did not screen out KLA in HK‐2 cells, this did not exclude the possibility that KLA could also exert a renal protection function similar to KLB.^[^ ^56^, ^57^ ^]^ In other words, Smg may alleviate renal injury in DKD patients by upregulating both KLA and KLB.

KLB may restore kidney function through AMPK phosphorylation and ferroptosis inhibition, and the results of cellular transcriptomics support this contention. Activation of AMPK could rewire lipid synthesis, iron metabolism, and energy metabolism, and boost antioxidant response for renal protection. Some work reported that FGF21 could activate AMPK signaling,^[^ ^92^ ^]^ which orchestrated the renal protection of KLB. Our findings revealed that the extent of reduction of serum KLB in DKD patients was more evident than that of KLA, indicating KLB is a more sensitive marker of kidney injury in response to treatment with Smg. Apart from Smg, other types of GLP‐1RAs (e.g., Lrg and Dlg) could also up‐regulate KLB, indicating that GLP‐1RAs may have a common ability of ferroptosis inhibition under a diabetic milieu.

Although the development and progression of DKD also involve other cell death pathways (e.g., necroptosis and pyroptosis), ferroptosis is considered the most critical pathway in DKD.^[^ ^93^, ^94^ ^]^ Receptor‐interacting protein‐1 and 3 and mixed lineage kinase domain‐like pseudokinase are primary regulators in the necroptosis signaling cascade.^[^ ^95^ ^]^ Dysregulation of necroptosis has been widely implicated in various kidney diseases.^[^ ^96^ ^]^ It has been reported that necroptosis might propagate cell death through ferroptosis, leading to cell lysis and inflammatory response.^[^ ^97^ ^]^ Meanwhile, ferroptosis can augment pyroptosis mediated by gasdermin D (GSDMD), accompanied by the release of pro‐inflammatory signals such as IL‐1β and IL‐18.^[^ ^98^ ^]^ Upon inflammasome stimulation, the activated inflammatory caspases process GSDMD into an N‐terminal fragment of GSDMD (GSDMD‐N) that oligomerizes in the plasma membrane, resulting in pore formation and pyroptotic lysis of cells.^[^ ^99^ ^]^ As a master regulator of lipid peroxidation and ferroptosis, the selenoprotein GPX4 can suppress the activation of inflammatory caspases (e.g., caspase 1 and 11) and block the phospholipase C gamma 1 mediated GSDMD activity.^[^ ^100^ ^]^ Therefore, Smg‐mediated ferroptosis inhibition may also suppress necroptosis and pyroptosis, during which the up‐regulated GPX4 by KLB/AMPK/NRF2 pathway is presumed as the major player.

This study not only revealed the mechanism by which GLP‐1RAs protected the kidney in DKD but also highlights their potential in managing a variety of diseases. First, patients with diabetes often suffer from multiple diabetic comorbidities besides kidney injury, such as cardiovascular disease, retinopathy, neuropathy, and foot ulcer, for which GLP‐1RAs may be beneficial.^[^ ^101^ ^]^ In particular, the beneficial effects of GLP‐1RAs on cardiovascular disorder in type II diabetes patients have been well‐reported.^[^ ^102^, ^103^ ^]^ A recent report also demonstrated that GLP‐1RAs could reduce cardiometabolic complications by reducing inflammation.^[^ ^104^ ^]^ Second, ferroptosis is linked with many pathophysiological conditions, including stroke, neurodegenerative diseases, ischemia‐reperfusion injury, age‐related macular degeneration, and doxorubicin cardiomyopathy.^[^ ^105^, ^106^, ^107^ ^]^ Iron chelation, radical trapping, and lipoxygenase inhibition are the canonical ferroptosis suppression approaches.^[^ ^108^ ^]^ However, these treatments often show the limitations of poor aqueous solubility, short half‐life, and unsatisfactory pharmacokinetics. Moreover, these treatments usually target a single ferroptotic signaling pathway. In contrast, GLP‐1RAs can simultaneously modulate iron metabolism, fatty acid synthesis, and antioxidant enzymes, exhibiting potential in managing the aforementioned diseases as pleiotropic ferroptosis inhibitors. The safety and tolerability of GLP‐1RAs have been proved with multiple dose forms available in the market. Targeting Klotho may represent a promising approach for potent ferroptosis inhibition and disease management, especially when messenger RNA (mRNA) delivery systems appear to be an innovative therapeutic platform for preventing and treating various diseases.^[^ ^109^ ^]^ Altogether, our results elucidate a novel molecular mechanism by which GLP‐1RAs can protect diabetic kidneys from ferroptosis‐mediated injury.

## 4 Experimental Section

### Patients

Thirty patients were selected with informed patient consent based on the following criteria:^[^ ^110^, ^111^ ^]^ (1) DKD induced by Type II diabetes mellitus, characterized with UACR ≥30 mg g^−1^, or eGFR at 30–60 mL min^−1^ 1.73^−1^ m^2^ for more than three months; (2) 18–75 years old; (3) HbA1c level at 7.0–10.0%; (4) Constant treatment with no more than 3 oral hypoglycemic agents for over three months; (5) The patients were diagnosed with dyslipidemia with or without taking the lipid‐regulating medicines; (6) Maximum tolerated dose of renin‐angiotension system inhibitor for more than three months. The exclusion criteria were designed as follows: (1) Serum calcitonin concentration ≥50 ng L^−1^; (2) Personal or family history of medullary thyroid carcinoma or multiple endocrine neoplasia syndrome type II; (3) Abnormal liver function with alanine aminotransferase and aspartate aminotransferase three times higher than the upper limit of standard value; (4) Severe nondiabetic kidney disease, or a recent history of dialysis for acute kidney failure, or kidney transplant; (5) Chronic pancreatitis or idiopathic acute pancreatitis; (6) Uncontrolled hypertension; (7) Severe diabetic complications such as diabetic ketoacidosis, hyperosmotic hyperglycaemia syndrome and lactic acidosis, and severe cardiovascular & cerebrovascular diseases (e.g., stroke, transient ischemic cerebral attack, acute coronary syndrome, or heart failure) in the past three months prior to the screening visit; (8) Use of GLP‐1RAs within three months prior to screening; (10) Pregnancy/lactation; (11) Any contraindication for MRI examination (e.g., pacemaker, metal prosthesis, and severe claustrophobia).

### DKD Patient Treatment

The sample size was determined according to the results of Ahmed M. Shaman et al. and eventually the eligible 30 DKD patients were randomly and equally assigned to receive insulin detemir or semaglutide via the random number table method.^[^ ^112^ ^]^ One group of patients receives insulin (Levemir U‐100, Novo Nordisk A/S, Bagsværd, Denmark) treatment, i.e., DKD/Ins. The patients in another group were given Smg (Novo Nordisk A/S, Bagsværd, Denmark) treatment, i.e., DKD/Smg. Before the fixed‐dose treatment, both groups of patients were subject to a dose escalation regimen. Regarding the DKD/Smg group, the patients were subcutaneously given 0.25 mg Smg once a week, and the dose was elevated to 0.5 mg (the maintenance dose) once a week for 4 weeks. Regarding the DKD/Ins group, the patients received a starting dose of 0.2 U kg^−1^ per day via subcutaneous injection. The dose was escalated by 2 U per person every four days to enable the fasting blood glucose within 4.4‐6.1 mM. After four weeks, the maintaining dose was fixed, and the patients were treated for another 24 weeks. The dosing frequency was once a week (DKD/Smg) and once a day (DKD/Ins), respectively. During the whole course of treatment, previous oral hypoglycemic therapy was continued for all patients. Among the 15 patients within the DKD/Smg group, two patients quitted during treatment.

### Biomarker Analysis in Patients

At the end of maintenance dose treatment (i.e., after 28 weeks), the blood and urine samples were collected from the DKD/Smg (*n* = 13) and DKD/Ins patients (*n* = 15). The concentration of KLB in the serum and urine was quantified by the ELISA kit. Selected ferroptosis biomarkers (GSH, MDA, and 4‐HNE) in the blood and urine were also determined by commercial assay kit, and the protein level was determined by the BCA assay. 

### Magnetic Resonance Imaging

Three DKD/Ins patients and three DKD/Smg patients were selected for MRI analysis. MRI scanning was conducted at the time of enrollment (i.e., baseline) and 28 weeks post Smg/Ins treatment using a PhilipsIngenia 3.0T superconducting MRI (Royal Dutch Philips Electronics Ltd, Amsterdam, The Netherlands) scanner coupled with a 32‐channel phased array body coil. The instrument was performed by professional doctors and technicians, and no food or water was allowed for 3 h before the examination. The patient respiratory rate was fixed at 18–20/min. The T1WI, T2WI, ASL, BOLD, mDixon‐Quant, and DTI were performed with the following parameters. T1WI: transverse position, TR 11 ms, TE 2.3 ms, FOV 380 mm × 294 mm, and slice thickness 6 mm; T2WI: transverse position, TR 1800 ms, TE 92 ms, FOV 360 mm × 360 mm, and slice thickness 6 mm. Regarding ASL, the pseudo‐continuous ASL tagging method was used to obtain the image. The breath‐holding scan was obtained at the end of inhalation in the oblique sagittal position. The scanning parameters were as follows: TR 3755 ms, TE 22 ms, TI 1200 ms, flip angle 90°, FOV 300 mm × 320 mm, and matrix 100 × 57. BOLD: coronal, breath‐holding scan, plane echo sequence of five echo times, TR 81 ms, TE 2.3 ms, slice thickness 5 mm, FOV 200 mm × 373 mm, and matrix 68 × 119. mDixon‐Quant: TR 5.7 ms, TE 0.97 ms, FOV 240 mm × 395 mm, slice thickness 6.5 mm and matrix 78 × 132. DTI: coronal, breath‐holding scan, TR 3000 ms, TE 39 ms, *b* = 0,400 s per mm^2^, FOV 223 mm × 223 mm, slice thickness 4 mm and matrix 112 ×112. The MRI images were analyzed as follows and single‐blind was suitable for MRI image information processing analysts. Post raw data import, six circular regions of interest (ROI) were manually delineated at the renal cortex's upper, middle, and lower poles on each side. Each ROI was 4–15 mm^2,^ depending on the kidney size and cortical thickness. The average value was taken as the measured blood flow RBF value. The change of RBF from the beginning to the 28th week was used as the index for ASL MRI. Likewise, the R2*, FF, and FA were the indices of BOLD, mDixon, and DTI MRI, respectively. The index change in the 28^th^ week against the starting point was calculated and compared. Regarding statistical analysis, the normality test was first carried out. The data were analyzed using the student's *t*‐test.

### Mice

A total of 40 eight‐week‐old male C57BL/6 mice (HFK Bioscience, Beijing, China) weighing 22–26 g was obtained and randomly divided into two groups: ten mice were as control (Ctl, *n* = 10) while thirty mice were used to establish the DKD mice model. Random numbers were generated using the standard = RAND() function in Microsoft Excel. All mice were housed in a pathogen‐free cage at ambient temperature under a 12 h light/dark cycle with free food and water access. The control mice were fed a regular diet (10% calories from fat, D12450B, Research Diet, America), whereas others were fed a high‐fat diet (HFD, 60% of calories from fat, D12492, Research Diets) during the whole course (Figure 2A). The HFD was employed coupled with low‐dose streptozotocin (STZ, 40 mg kg^−1^) to induce DKD in mice. Mice fed with HFD for ten weeks and with blood glucose levels higher than 16.7 mmol L^−1^ after 1‐week post five consecutive STZ exposure were considered diabetic, and then respectively received Smg (DKD/Smg, 60 µg kg^−1^, twice a week) or Fer‐1 (DKD/Fer‐1, 1 mg kg^−1^, daily) for eight weeks when the UACR in diabetic mice was higher than in the control mice, whereas the DKD group received the equivalent volume of normal saline. Each group contains eight mice following a previous experiment in the same laboratory, testing the same treatment under similar conditions on animals with the same characteristics. Regarding the overexpression of KLB in vivo, the NC and DKD mice were injected with adeno‐associated virus serotype 9 (AAV9)‐Klb (named AAV‐Klb) by tail vein with 200 µL of virus containing 2 × 10^11^ vg of vectors, while the empty vector (AAV‐Control) was injected into mice as a negative control group. The AAVs used above were packaged and purified by Genechem Co., LTD. (Shanghai, China).

### Biochemical Measurements

The blood glucose level of mice in four groups (Ctl, DKD, DKD/Smg, and DKD/Fer‐1) was monitored daily until the 16th week. The blood and urine samples were collected on the 16th week when the mice were sacrificed. Blood and urine samples were centrifuged for 15 min to collect the supernatant for further analysis using a biochemistry autoanalyzer. The blood samples were analyzed regarding creatinine, and blood urea nitrogen. Albumin, creatine, *N*‐acetyl‐*β*‐glucosaminidase, and transferrin were analyzed in the urine samples. The UACR was calculated accordingly.

### Histologic Evaluation

The kidney was excised on the 16th week and the kidney tissues were fixed using 4% (w/v) paraformaldehyde for 48 h, embedded in paraffin, and sectioned into four µm‐thick slices. Tissue slides were stained with H&E, Masson, and PAS after de‐paraffinization with xylene, rehydration with ethanol, and sealing with neutral balsam. Then, the kidney tissues were imaged via an optical microscope. The extent of renal injury was determined using the indices of glomerular hypertrophy, tubular damage, mesangial matrix expansion, and interstitial fibrosis.

### Immunohistochemical Analyses

The tissues were subject to fixing, embedding, cutting, mounting, deparaffinizing, rehydrating, heat‐mediated antigen retrieval, and blocking. Then, the tissue slices were incubated with primary antibodies against fibrosis biomarkers TGF‐β1, α‐SMA, E‐Cad, and vimentin at 4 °C for 12 h. The tissue slices were washed with PBS in triplicate and incubated with a goat anti‐rabbit IgG HRP polymer at 37 °C for 1 h. Subsequently, an HRP‐DAB system (Proteintech) was used to detect the immunoactivity, followed by counterstaining with hematoxylin and image recording using a light microscope.

### Immunofluorescence Staining

The immunofluorescence technique was employed to assess the expression and distribution of GLP‐1R in renal tissue. The tissues were subject to fixing, embedding, cutting, mounting, deparaffinizing, rehydrating, heat‐mediated antigen retrieval, and blocking. Then, the tissue slices were incubated with or without primary antibodies against GLP‐1R at 4 °C overnight. Afterward, the tissue slices were incubated with goat anti‐rabbit (IgG) secondary antibody with TRITC in the dark for 1 h, followed by nuclei staining with DAPI and captured the images by the fluorescence microscope.

### Cell Cultures

Human kidney proximal tubular cells (HK‐2, American Type Culture Collection, Rockville, MD) were cultured in DMEM medium (Gibco, Carlsbad, CA, USA) containing 5.5 mmol L^−1^ glucose, 10% fetal bovine serum (ScienCell, San Diego, CA, USA), 100 U mL^−1^ penicillin and streptomycin (Solarbio, Beijing, China) at 37 °C, 95% humidity, and 5% CO_2_. The cells cultured with 5.5 mM glucose were defined as the NG group, while the cells cultured in 35 mM glucose and 120 µM palmitic acid were named the HGL group. The HGL cells with a 400 nM semaglutide (Novo Nordisk, Denmark) were defined as the HGL/Smg group.

Mouse primary kidney tubular cells were isolated from C57BL/6 mice (4‐8 weeks old). Briefly, the renal cortex was separated, minced, and treated with 0.1% collagenase type II at 37 °C with gentle agitation for 20 min. The digested tissue was passed through a 70 µm cell strainer, and the filtrate was centrifuged at 150 *g* for 10 min to pellet the cells. Subsequently, the cell pellet was resuspended in complete growth medium (DMEM/F12 supplemented with 10% FBS, 1% penicillin/streptomycin) and then plated onto cell culture dishes and incubated at 37 °C in a humidified atmosphere containing 5% CO2. After 24 h, the medium was changed to remove non‐adherent cells. Once the cells reached 80–90% confluence, they were trypsinized and seeded in a 6‐well plate for experiments.

### Transcriptomic Analysis

The HK‐2 cells were seeded in 6‐well plates at a density of 5 × 10^5^ per well. The NG group was incubated in a medium containing 5.5 mM glucose. The medium in the HGL group contains 35 mM glucose and 120 µM palmitic acid (PA). The medium in the HGL/Smg group contains glucose (35 mM), PA (120 µM), and Smg (400 nM). After 48 h's incubation, the cells were trypsinized and centrifuged (500 *g*, 5 min) (*n* = 3). The re‐suspended cells in phosphate‐buffered saline (PBS) were centrifuged again (500 *g*, 5 min). The obtained pellets containing 5 × 10^6^ cells were mixed with TRIzol lysis reagent. Afterward, detailed RNA sequencing was performed by Applied Protein Technology (APTBIO, Shanghai, China), and the data generated from the Illumina platform were used for bioinformatics analysis.

### Cell Viability Assay

Cell viability was measured using the Cell Counting Kit‐8 (BS350B, Biosharp). Cells seeded in 96‐well plates were treated with various concentrations of the compounds and incubated with 10% CCK8 solution for 30 min at 37 °C. The absorbance was measured by the microplate reader (Synergy HT, Bio‐Tek) at 450 nm.

### Confocal Microscopy

The cytosolic and mitochondrial Fe^2+^ concentration in HK‐2 cells under different conditions (NG, HGL, and HGL/Smg) was analyzed by the FerroOrange (1 µM) and Mito‐FerroGreen (5 µM) probe, respectively. The intracellular ROS and lipid peroxides were determined using a canonical dichlorodihydro ‐fluorescein diacetate (DCFH‐DA, 20 µM) and Liperfluo probe (5 µM), respectively. The cells were incubated with these above probes at 37 °C for 30 min, and the nuclei were stained with DAPI (1 µg mL^−1^) for 10 min, followed by image recording using a Zeiss LSM 800 confocal microscope. The excitation wavelength was 543 nm, 505 nm, 488 nm, and 488 nm for FerroOrange, FerroGreen, DCFH‐DA, and Liperfluo, respectively. The fluorescence intensity was analyzed by Image J.

### Mitochondrial Morphology Analysis

The HK‐2 cells under different conditions (NG, HGL, and HGL/Smg) were fixed using 2.5% (w/v) glutaraldehyde solution in PBS (0.1 M, pH 7.4) at 4 °C for 3 h. Afterward, the cells were post‐fixed in 1% (w/v) osmium tetroxide solution in PBS (0.1 M, pH 7.4) at ambient temperature for 2 h, followed by dehydration in a series of increasing strength of ethanol (50–100%, v/v), embedding in epoxy resin, and curing at 60 °C for 48 h. Then thin sections (50 µm) were cut using the Leica EM UC‐7 microtome, followed by staining with 2% (w/v) uranyl acetate and 0.4% (w/v) lead citrate, and image recording using a transmission electron microscope (HT7700‐SS; HITACHI, Tokyo, Japan).

### Fe^2+^, GSH, and MDA Assays

Cells collected from 6‐well plates were centrifuged, and washed, and then added 200 uL PBS for sonication fragmentation to obtain cell lysate. In each group, 20 mg kidney tissue was excised and homogenized by vibrating homogenizer with tissue lysates and then centrifuged for 15 min to collect the supernatants to measure the level of Fe^2+^ (DIFE‐250, BioAssay), GSH (BC1175, Solarbio) and MDA (BC0025, Solarbio). The collected supernatants were transferred into a 96‐well plate and measured the optical densities of Fe^2+^, GSH, and MDA at 590, 412, 600, and 532 nm, respectively. Afterward, the concentrations of Fe^2+^ and GSH were calculated according to the standard curve, and the level of MDA was calculated according to the manufacturer's instructions. The total protein level was determined by the bicinchoninic acid (BCA) method. The data were normalized against the protein level (*n* ≥ 3).

### ELISA Assay

The levels of 4‐HNE, AA, and AdA in supernatants of HK‐2 cell and kidney tissue were measured using ELISA kits (Table S2, Supporting Information) according to the manufacturer's instruction. In addition, inflammation and fibrosis biomarkers (IL‐1β, TNF‐α, IL‐6, MCP‐1, IL‐10, and TGF‐β1) of kidney tissue were measured using ELISA kits. The total protein level was determined by the bicinchoninic acid (BCA) method. The data were normalized against the protein level (*n* ≥ 3).

### NAD(P)^+^ and NAD(P)H Assay

The levels of NAD(P)^+^ and NAD(P)H (S0175, S0179, Beyotime) in supernatants of HK‐2 cells under different conditions were quantified by the corresponding colorimetric assay kit following the manufacturer's protocols.

### Western Blot Analysis

Extracted the proteins of HK‐2 cells and kidney tissues by using RIPA lysis buffer (R0010, Solarbio), and detected the concentrations by using a BCA assay kit. The samples containing equal amounts of proteins (30‐50 µg) were subject to SDS‐polyacrylamide gel electrophoresis and then transferred to the nitrocellulose membrane. Afterward, the membranes were blocked with 5% skim milk at ambient temperature for 1 h, incubated with indicated diluted primary antibodies at 4 °C for 12 h, and then HRP‐conjugated secondary antibodies at ambient temperature for 1 h. The protein bands were developed using ECL western blotting detection reagent and imaged by Tanon 6600 Imager (Shanghai, China). The protein band intensity was analyzed by Image J (*n* ≥ 3).

### qRT‐PCR

The total RNA of HK‐2 cells under different conditions was extracted and purified by using the HP Total RNA Kit (Omega Bio‐Tek, Norcross, GA, USA). The cDNA was synthesized using a reverse transcription kit (Vazyme, Nanjing, China). Quantitative PCR (qPCR) reactions were performed in 10 µL of final volume using the SYBR Green qPCR Mix (ABclonal) and the CFX96 Real‐Time PCR detection system (Bio‐Rad). The relative amount of the target genes was standardized against GAPDH or β‐actin and determined using the 2^−ΔΔCT^ method (*n* ≥ 3).

### siRNA and Plasmids Transfection

The RNA interference technique was employed to knock down KLB and CREB, respectively (*n* ≥ 3). In brief, 50 µL siRNA (siKLB, siCREB, siCtl) at a concentration of 2 µM was mixed with the cationic lipofectamine 3000 (50 µL, dilution: 10 times) to form a polyplex in Opti‐MEM serum‐free media. The HK‐2 cells (900 µL) were then incubated with the polyplex (100 µL) in a 12‐well plate under standard cell culture conditions for 6 h, followed by the replacement of fresh culture medium. Then, the cells were cultured under HGL/Smg or NG conditions. After 48 h, the cells were subjected to western blotting of target proteins and mRNA quantification of the corresponding genes.

To assess the rescue effect of plasmid KLB delivery on Ave‐treated HK‐2 cells, the cells were transfected with polyplexes containing either empty plasmid vector (OE‐Ctl) or over‐expression plasmid of KLB (OE‐KLB, 1.0 µg) for 24 h. The production of polyplexes was as follows. In short, 1.0 µg plasmid DNA (OE‐Ctl and OE‐KLB) were mixed with the P3000 reagent (2 µL µg^−1^ DNA)) and cationic lipofectamine 3000 (50 µL, dilution: 10 times) to form the polyplexes in Opti‐MEM serum‐free media. Then, the HK‐2 cells dispersed in 900 µL normal culture medium were incubated with the above polyplex dispersion (100 µL) in a 12‐well plate under standard cell culture conditions.

### Statistical Analysis

Data were presented as mean ± standard error from at least three biological replicates of experiments. Statistical comparison were performed using Student's *t*‐test or one‐way ANOVA with a Tukey post‐hoc analysis. Significance levels are as follows: *p < 0.05, **p < 0.01, ***p < 0.001; ns indicates non‐significant. All statistical analyses were performed using either GraphPad Prism 9.0 or SPSS 25.0 software.

### Ethics Approval Statement and Consent to Participate

This clinical study was with ethical approval by the Ethics Committee of the Chu Hsien‐I Memorial Hospital of Tianjin Medical University for clinical research (ZXYJNYYsMEC2023‐41) and was registered at chictr.org.cn under the number ChiCTR2400080751. All the participants provided written informed consents before inclusion in this study. The animal experiments were approved by the ethical committee of Tianjin Medical University (Institutional Animal Care and Use Committee Issue No. DXBYY‐IACUC‐2022061) and followed the ARRIVE reporting guidelines.^[^ ^113^ ^]^

## Conflict of Interest

The authors declare no conflict of interest.

## Author Contributions

S.T., S.Z., and W.W. contributed equally to this work. Y.Z. and P.Y. performed conceptualization. S.T., S.Z., W.W., Y.L., T.W., H.S., A.A‐N.‐W. performed the experiment. P.Y. and S.Z. performed funding acquisition. Y.Z., P.Y., H.S., C.W., and X.L. performed data analysis. Y.Z., S.T., S.Z., P.Y., and X.L. performed writing and correction.

## Supporting information



Supporting Information

## Data Availability

The data that support the findings of this study are available from the corresponding author upon reasonable request.
