# Supplementary material for: GLP‐1 Receptor Agonists Alleviate Diabetic Kidney Injury via β‐Klotho‐Mediated Ferroptosis Inhibition
Source: Adv Sci (Weinh). 2024 Dec 4;12(4):2409781. doi: 10.1002/advs.202409781 (PMC11775532; doi:10.1002/advs.202409781)
Supplement: Supplementary file 1 — Supporting Information [file ADVS-12-2409781-s001.docx]

GLP-1 Receptor Agonists Alleviate Diabetic Kidney Injury via β-Klotho-Mediated Ferroptosis Inhibition

*Shasha Tian^#^, Saijun Zhou^#^, Weixi Wu^#^, Yao lin, Tongdan Wang, Haizhen Sun, A-Shan-Jiang A-Ni-Wan, Yaru, Li, Chongyang Wang, Xiaogang Li, Pei Yu* and Yanjun Zhao**

S. Tian, S. Zhou, W. Wu, Y. Lin, T. Wang, H. Sun, A. ANW, P. Yu

NHC Key Laboratory of Hormones and Development, Chu Hsien-I Memorial Hospital and Tianjin Institute of Endocrinology

Tianjin Key Laboratory of Metabolic Diseases, Tianjin Medical University, Tianjin 300134, China

Email: [peiyu@tmu.edu.cn](mailto:peiyu@tmu.edu.cn)

Y. Li, Y. Zhao

School of Pharmaceutical Science & Technology, Tianjin Key Laboratory for Modern Drug Delivery & High Efficiency

Faculty of Medicine, Tianjin University, Tianjin 300072, China

Email: [zhaoyj@tju.edu.cn](mailto:peiyu@tmu.edu.cn)

C. Wang

School of Life Sciences, Peking University, Beijing 100871, China

X. Li

Department of Internal Medicine, Mayo Clinic, Rochester, MN 55901, USA

P. Yu

Nephropathy & Blood Purification Department, The Second Hospital of Tianjin Medical University, Tianjin 300134, China

S. Tian

Department of Nephrology, The Fifth Hospital of Shanxi Medical University (Shanxi Provincial People's Hospital), Taiyuan, Shanxi 030000 China

# These authors contributed equally: Shasha Tian, Saijun Zhou, Weixi Wu.

Excluded (n=59)

¨  Not meeting inclusion criteria (n= 30)

- HbA1c level over 10.0% (8)
- Uncontrolled hypertension (7)
- more than 3 oral hypoglycemic agents (4)
- severe cardiovascular & cerebrovascular diseases (3)
- Severe nondiabetic kidney disease (3)
- abnormal liver function (3)
- DKD induced by Type I diabetes mellitus (2)

¨  Declined to participate (n=29)

Enrollment

Assessed for eligibility (n=89)

Randomized (n=30)

.

Allocated to DKD/Ins group (n=15)

Received allocated intervention (n=15)

Did not receive allocated intervention (give reasons) (n=0)

Allocation

Allocated to DKD/Smg group (n=15)

Received allocated intervention (n=15)

Did not receive allocated intervention (give reasons) (n=0)

¨ Did not receive allocated intervention (give reasons) (n= )

Follow-Up

Lost to follow-up (n=1)

Discontinued intervention (n=1)

- Fasting blood glucose was not controlled

Lost to follow-up (give reasons) (n=0)

Discontinued intervention (give reasons) (n=0)

Analysis

Analysed (n=13)
Excluded from analysis (n=0)

Analysed (n=15)

Excluded from analysis (n=0)

**Figure S1.** Participant Flow Diagram. Based on the CONSORT (Consolidated Standards of Reporting Trials) diagram (adapted from <http://www.consort-statement.org/>).


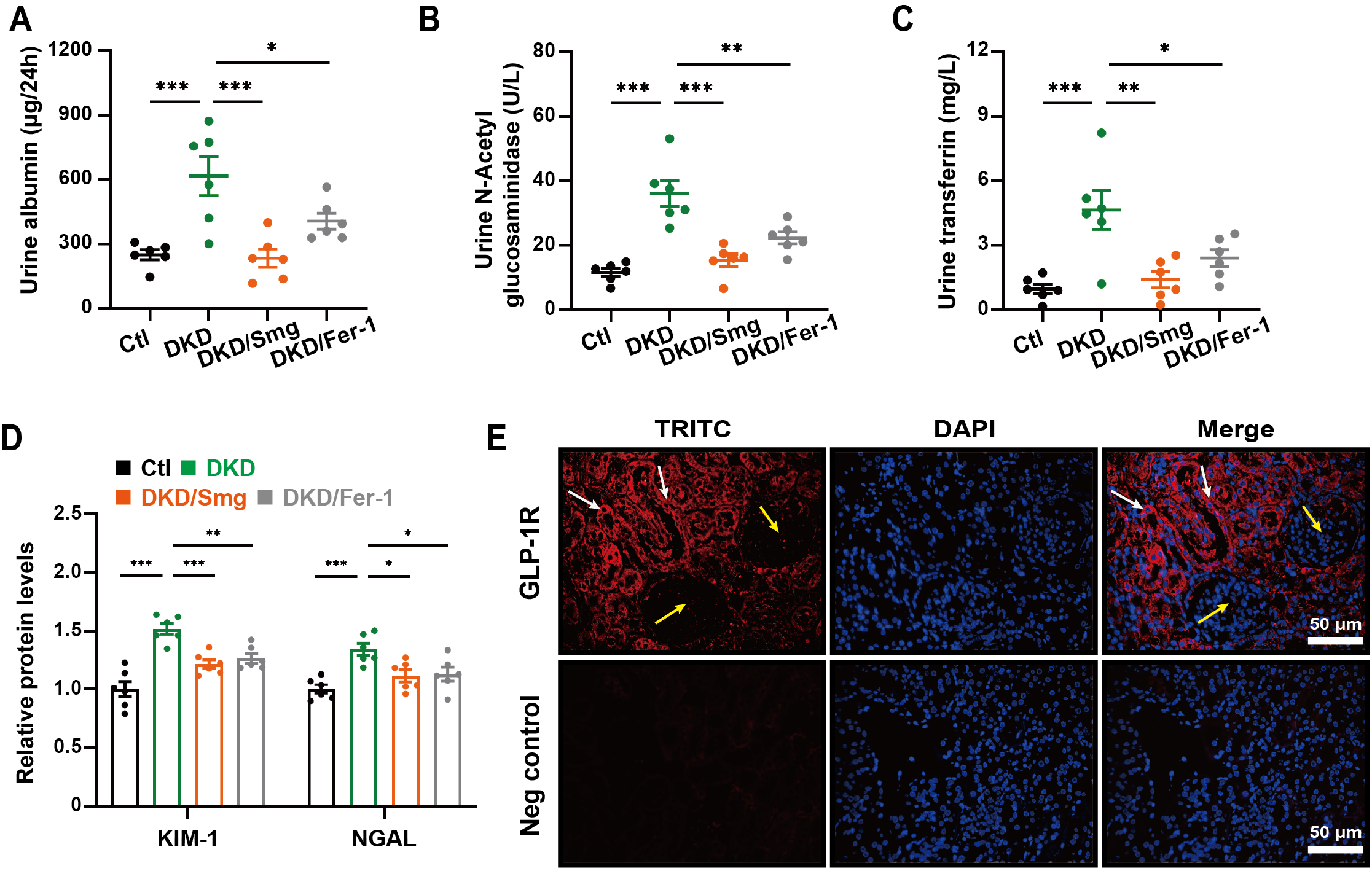


**Figure S2.** The treatment of Smg and ferroptosis inhibition relieves kidney injury in diabetic mice. A-C) The concentration of kidney injury biomarkers such as urinary albumin, N-acetyl-β-glucosaminidase (NAG), and transferrin in four mice models (Ctl, DKD, DKD/Smg, and DKD/Fer-1). D) Immunoblot quantification of KIM-1, NGAL in kidney tissues of mice treated by different samples. E) Immunofluorescence images of the expression and location of GLP-1R (red fluorescence signals) in kidney tissues, DAPI staining shows cell nuclei (blue). KIM-1: Kidney injury molecule 1; NGAL: Neutrophil gelatinase-associated lipocalin. Data are presented as mean ± standard error (*n* ≥ 6). Statistical comparison was performed using one-way ANOVA with a Tukey post-hoc analysis, * *p* < 0.05, ** *p* < 0.01, *** *p* < 0.001.


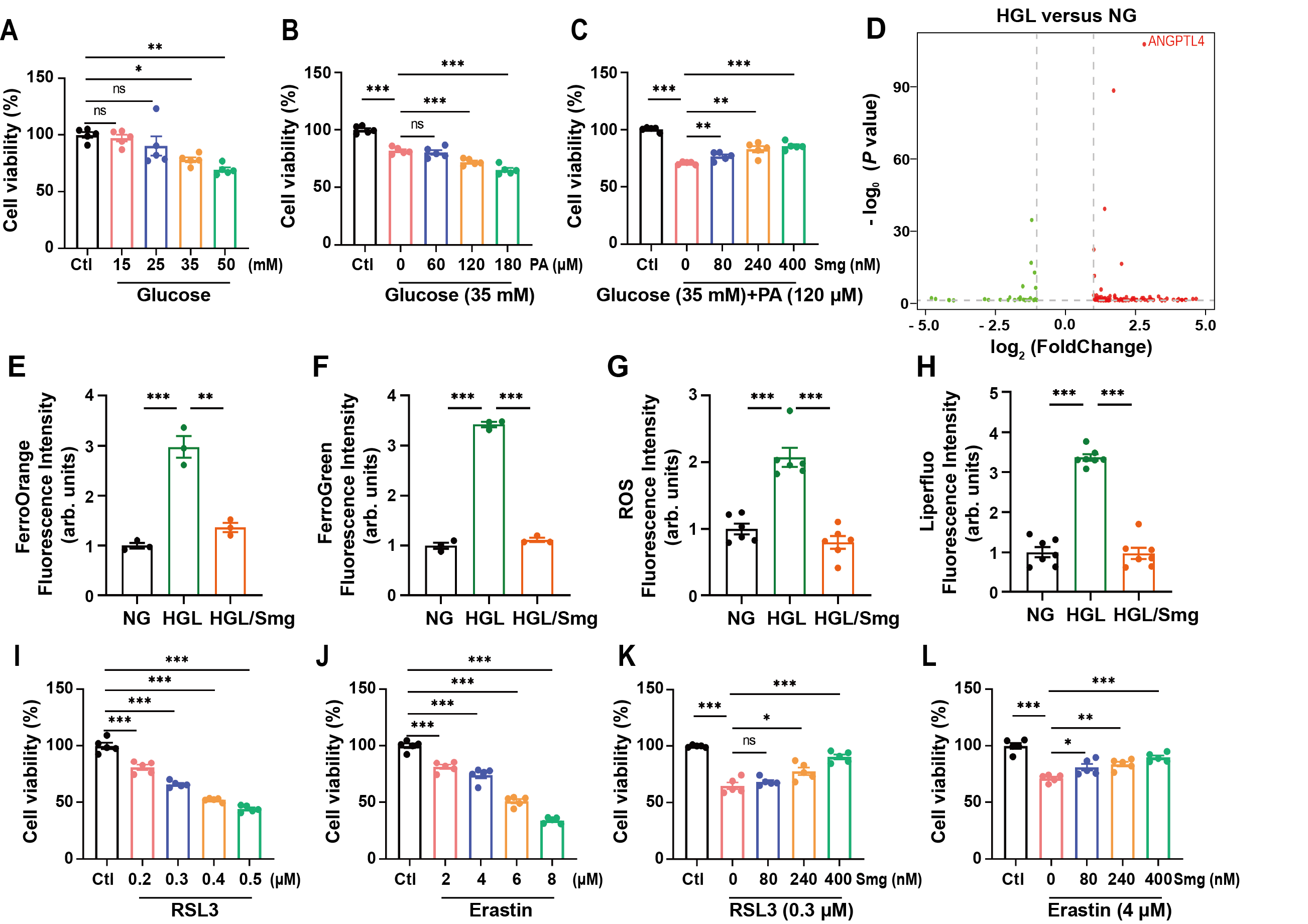


**Figure S3.** Smg rescues the HK-2 cells under ferroptotic stress. A) The viability of HK-2 cells in response to glucose at different doses. B) The viability of HK-2 cells in response to glucose (35 mM) and palmitic acid (PA) at different doses. C) The influence of Smg on the viability of HGL-treated HK-2 cells. D) Volcano plot showing the upregulated ANGPTL4 in HK-2 cells incubated with NG and HGL. E-H) The fluorescence intensity of the FerroOrange, FerroGreen, DCFH, and Liperfluo probes in HK-2 cells treated by NG (5.5 mM glucose), HGL (35 mM glucose and 120 µM palmitic acid/PA), or HGL/Smg (HGL plus Smg). I, J) The viability of HK-2 cells in response to RSL3 or Erastin at different doses. K, L)The influence of Smg on the viability of HK-2 cells in the presence of 0.3 µM RSL3 or 4 µM Erastin. All of these assays were performed after the HK-2 cells culturing for 48 hours under different conditions. ANGPTL4, angiopoietin-like 4. Data are presented as mean ± standard error (*n* ≥ 3). Statistical comparison was performed using one-way ANOVA with a Tukey post-hoc analysis, * *p* < 0.05, ** *p* < 0.01, *** *p* < 0.001.

**
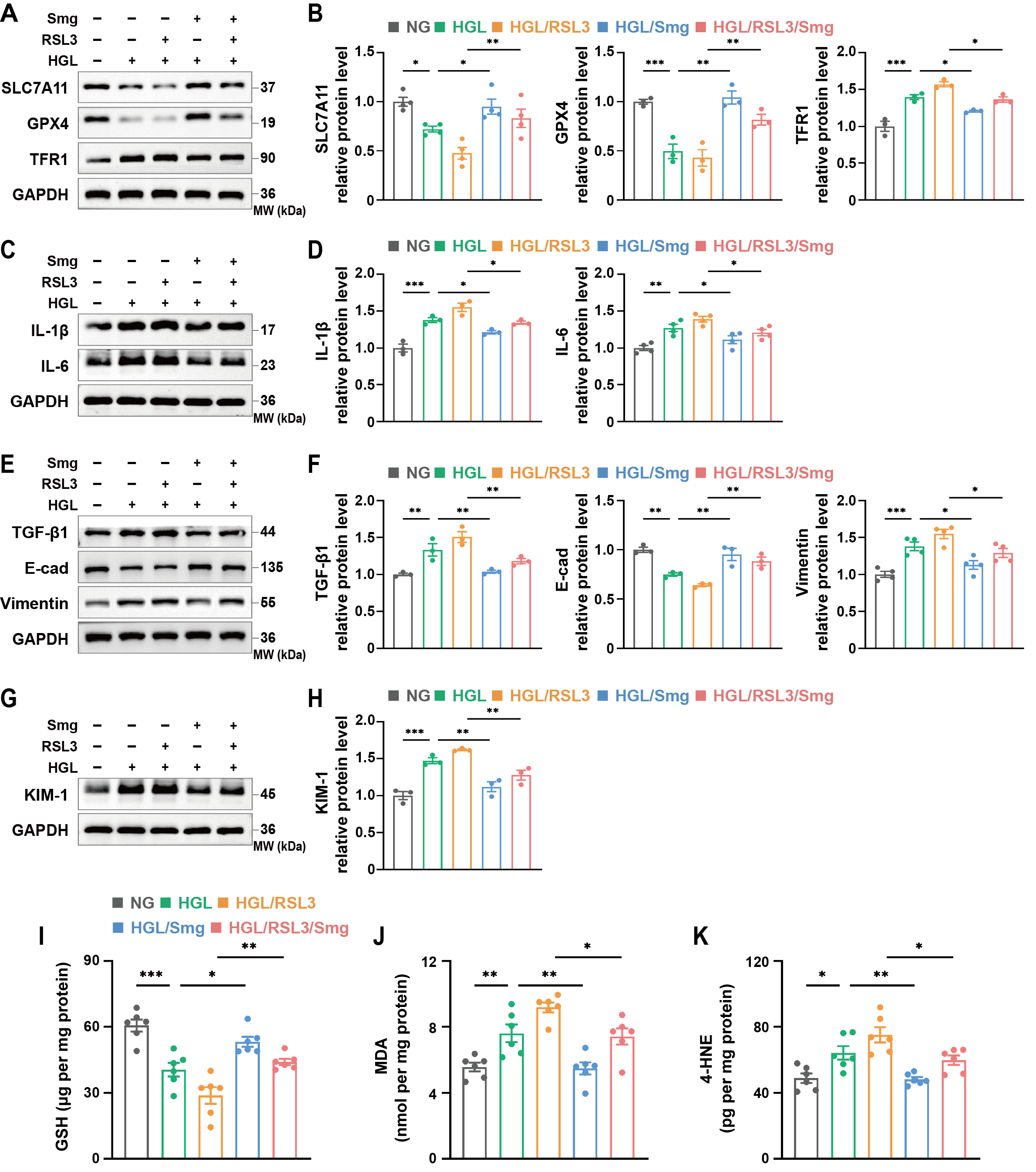
Figure S4.** Smg alleviates inflammation, fibrosis, and kidney damage via ferroptosis inhibition in primary renal tubular cells under HGL conditions. A, B) Immunoblot analysis and quantification of proteins associated with ferroptosis of primary renal tubular cells in the indicated groups. C, D) Immunoblot analysis and quantification of proteins associated with inflammation of primary renal tubular cells in the indicated groups. E, F) Immunoblot analysis and quantification of proteins associated with fibrosis of primary renal tubular cells in the indicated groups. G, H) Immunoblot analysis and quantification of proteins associated with kidney injury of primary renal tubular cells in the indicated groups. I - K) Quantification of intracellular GSH, MDA, and 4-HNE. Primary renal tubular cells were cultured in NG: 5.5 mM glucose; HGL: 35 mM glucose and 120 µM palmitic acid/PA; HGL/Smg: HGL plus 400 nM Smg; ferroptosis inducers: 0.3 µM RSL3. All of these assays were performed after the primary renal tubular cell culturing for 48 hours under different conditions. SLC7A11, solute carrier family 7 member 11; GPX4, glutathione peroxidase 4; IL-1β, interleukin-1β; IL-6, interleukin-6; TGF-β1, transforming growth factor-beta 1; E-cad, E-cadherin; KIM-1: Kidney injury molecule 1; GSH: glutathione; MDA: malondialdehyde; 4-HNE: 4-hydroxynonenal. Data are presented as mean ± standard error (*n* ≥ 3). Statistical comparison was performed using one-way ANOVA with a Tukey post-hoc analysis, * *p* < 0.05, ** *p* < 0.01, *** *p* < 0.001.


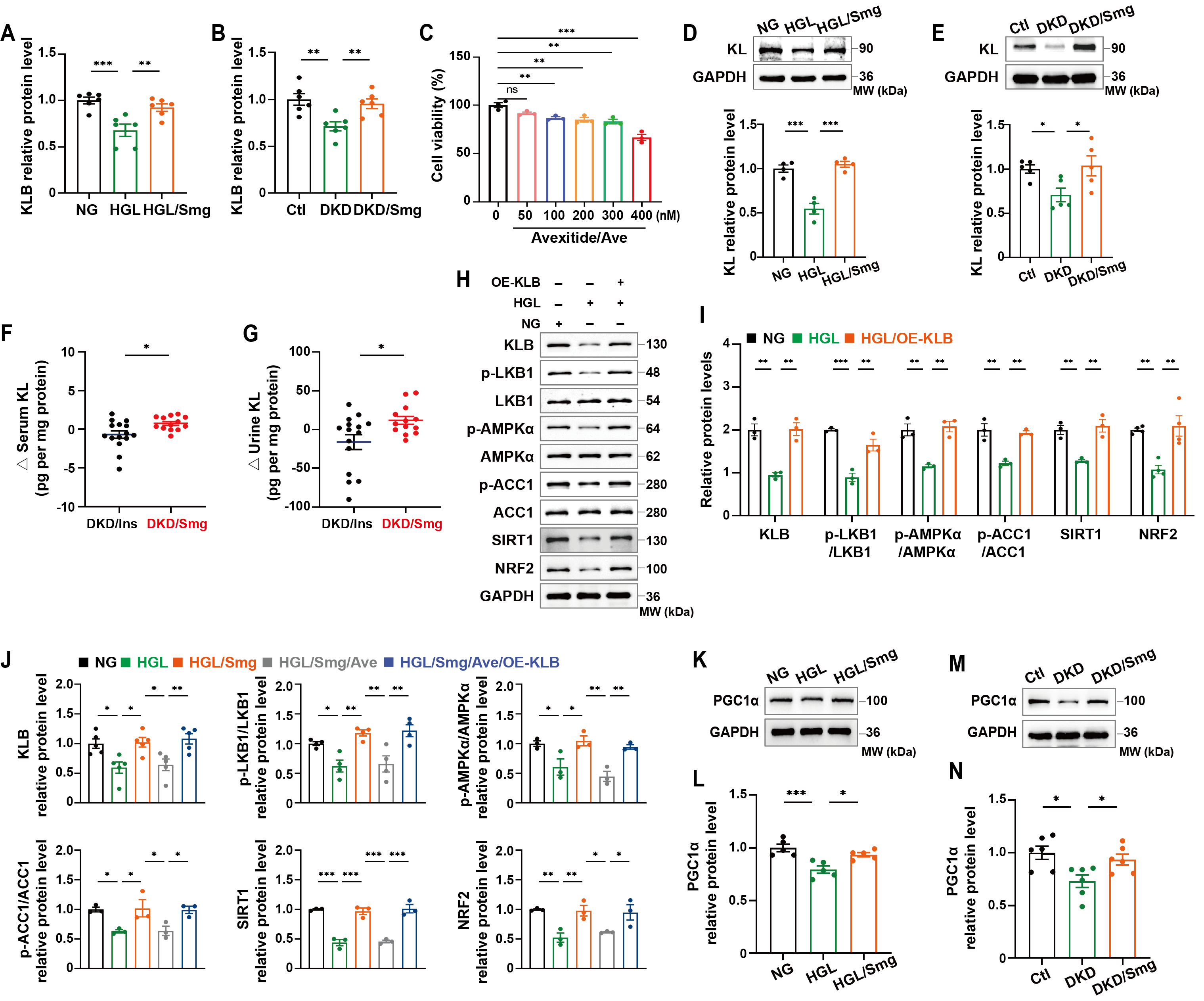


**Figure S5**. Smg regulates the AMPK signaling pathway via KLB under HGL conditions. A, B) Immunoblot quantification of KLB in vitro and vivo experiments in the indicated groups. C) The viability of HK-2 cells in response to Avexitide (Ave), the inhibitor of glucagon-like peptide-1 (GLP-1) receptor. D, E) Immunoblot analysis of KL protein expression in *vitro* and *vivo* experiments under diabetic conditions and combined with Smg treatment. F, G) The changes of serum and urine concentration of soluble α-Kloth in DKD/Ins and DKD/Smg patients. H, I) Immunoblot quantification of KLB and proteins associated with AMPK signaling pathway in HK-2 cells treated by NG, HGL, or HGL/OE-KLB. J) Immunoblot quantification of KLB and proteins associated with AMPK signaling pathway in HK-2 cells of the indicated groups. K-N) Immunoblot analysis and quantification of PGC-1α in vitro and vivo experiments in the indicated groups. HK-2 cells were cultured in NG: 5.5 mM glucose; HGL: 35 mM glucose and 120 µM palmitic acid/PA; HGL/Smg: HGL plus 400 nM Smg; HGL/Smg/Ave: HGL plus Smg and 300 nM Avexitide/Ave; OE-KLB: 1.0 μg mL^−1^ plasmid DNA of KLB. All of these assays were performed after the HK-2 cell culturing for 48 hours under different conditions. PGC-1α: proliferator-activated receptor gamma coactivator 1α. Data are presented as mean ± standard error (*n* ≥ 6). Statistical comparison was performed using one-way ANOVA with a Tukey post-hoc analysis, **p* < 0.05, ***p* < 0.01, ****p* < 0.001, ns indicates no significant difference.

**
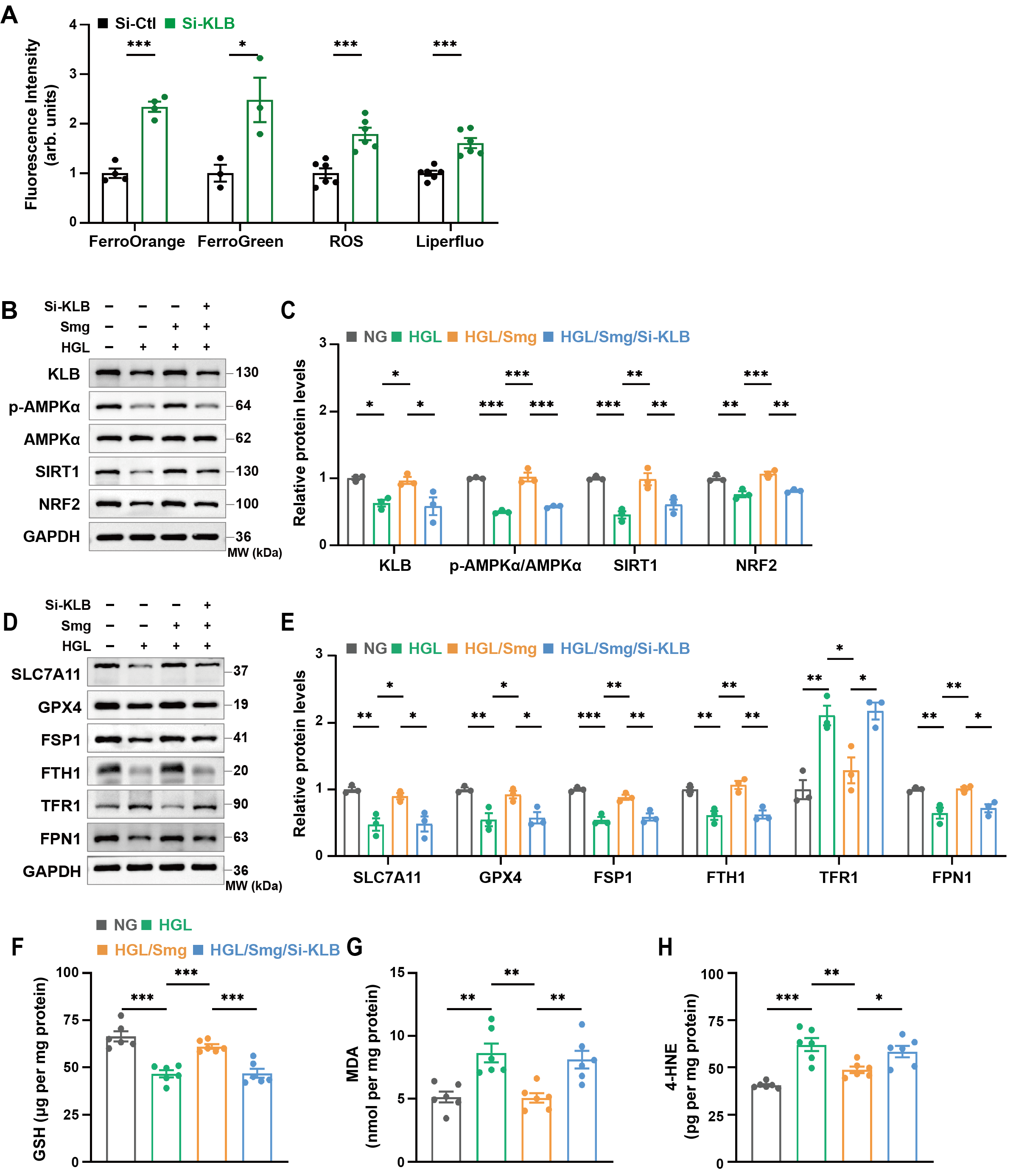
Figure S6.** Knockdown of KLB counteracts the action of Smg on ferroptosis under HGL/Smg condition in HK-2 cells and primary renal tubular cells. A) The fluorescence intensity of the FerroOrange probe (cytosolic Fe^2+^), FerroGreen probe (mitochondrial Fe^2+^), DCFH probe (reactive oxygen species/ROS), and Liperfluo probe (lipid peroxide) post KLB knockdown in HK-2 cells. B, C) Immunoblot quantification of KLB and proteins associated with AMPK signaling pathway in primary renal tubular cells treated by NG, HGL, HGL/Smg, or HGL/Smg/Si-KLB. D, E) Immunoblot quantification of proteins related to ferroptosis in primary renal tubular cells treated by NG, HGL, HGL/Smg, or HGL/Smg/Si-KLB. F-H) Quantification of intracellular GSH, MDA, and 4-HNE. HK-2 cells or primary renal tubular cells were cultured in NG: 5.5 mM glucose; HGL: 35 mM glucose and 120 µM palmitic acid/PA; HGL/Smg: HGL plus 400 nM Smg; HGL/Smg/Si-KLB: HGL and 100 nM si-RNA of KLB. These assays were performed after the HK-2 cells or primary renal tubular cells were cultured for 48 hours under different conditions. Data are presented as mean ± standard error (*n* ≥ 3). Statistical comparison was performed using unpaired two-tailed Student’s t-test (A) or one-way ANOVA with a Tukey post-hoc analysis, **p* < 0.05, ***p* < 0.01, ****p* < 0.001.


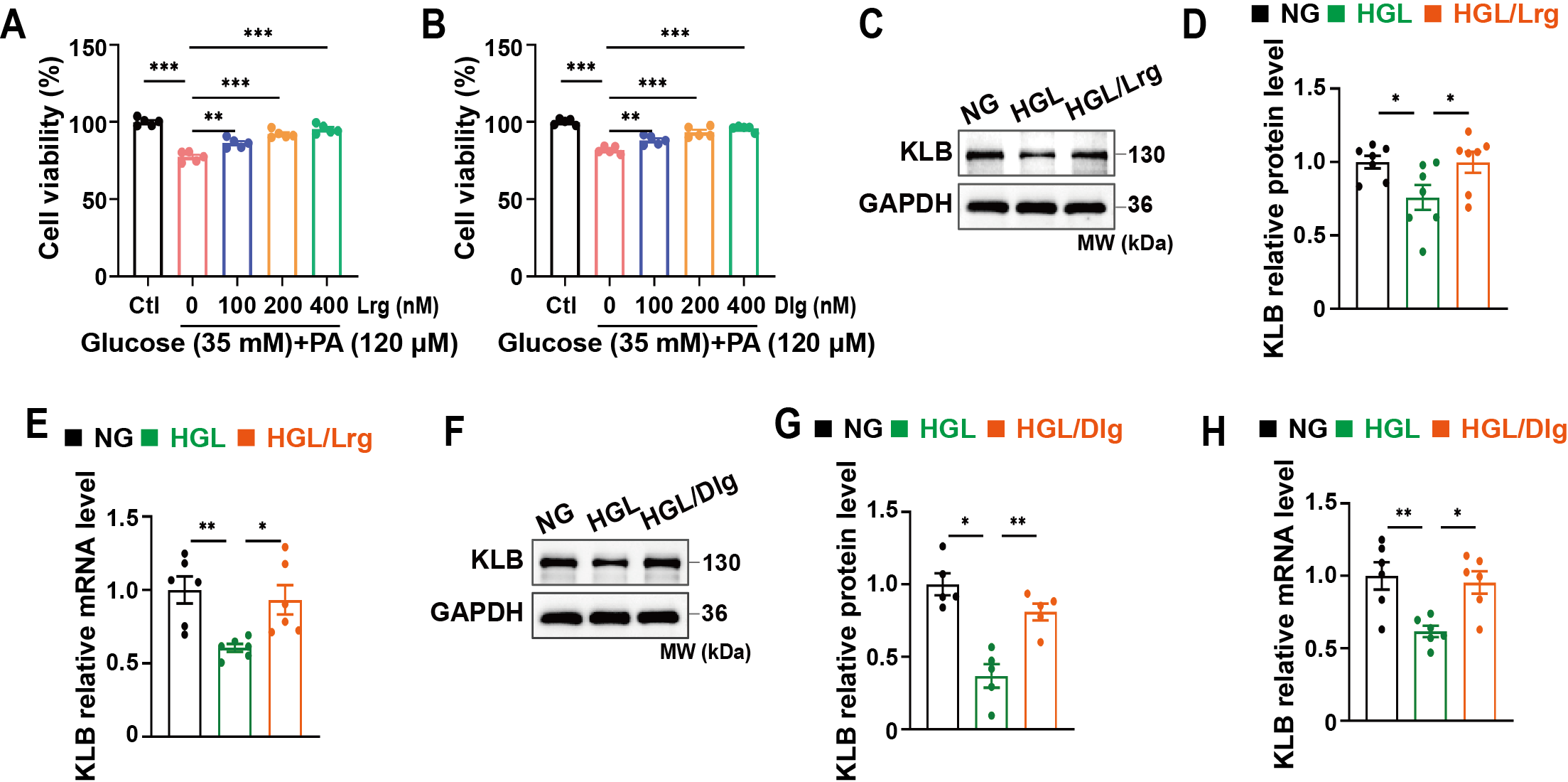


**Figure S7**. Liraglutide (Lrg) or dulaglutide (Dlg) increases KLB in HK-2 cells under HGL conditions. A) The influence of Lrg on the viability of HGL-treated HK-2 cells. B) The influence of Dlg on the viability of HGL-treated HK-2 cells. C) Immunoblotting of lysates of HK-2 cells with or without HGL and Lrg. D, E) Quantification of protein band intensity and mRNA of KLB post treatment by HGL or HGL/Lrg. F) Immunoblotting of lysates of HK-2 cells with or without HGL and Dlg. G, H) Quantification of protein band intensity and mRNA of KLB post treatment by HGL or HGL/Dlg. HK-2 cells were cultured in NG: 5.5 mM glucose; HGL: 35 mM glucose and 120 µM palmitic acid/PA; HGL/Lrg: HGL plus 400 nM Lrg; HGL/Dlg: HGL plus 400 nM Dlg. All of these assays were performed after the HK-2 cells culturing for 48 hours under different conditions. Data are presented as mean ± standard error (*n* ≥ 3). Statistical comparison was performed using one-way ANOVA with a Tukey post-hoc analysis, **p* < 0.05, ** *p* < 0.01, ****p* < 0.001.

**
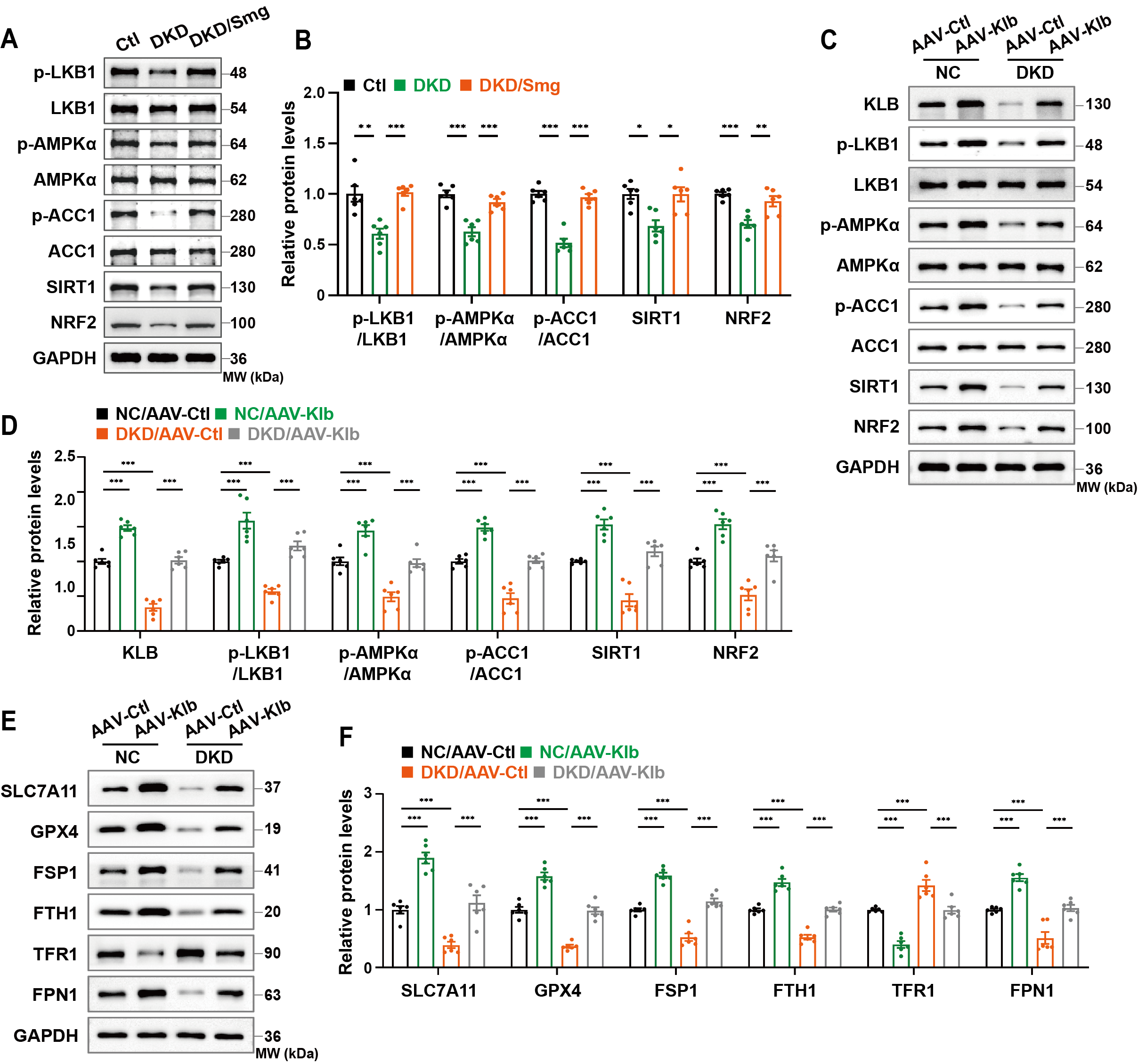
Figure S8**. Overexpression of KLB activates the AMPK signallig pathway and ameliorates ferroptosis in *vivo*. A, B) Immunoblot analysis and quantification of proteins associated with the AMPK pathway in the indicated groups. C, D) Immunoblot analysis and quantification of KLB and proteins associated with the AMPK pathway in the indicated groups. E, F) Immunoblot analysis and quantification of proteins associated with the ferroptosis in the indicated groups. KLB, β-Klotho; LKB1, liver kinase beta 1; AMPK, AMP-activated protein kinase; ACC, acetyl-CoA carboxylase; SIRT1, sirtuin 1; NRF2, nuclear factor erythroid 2-related factor 2; SLC7A11, solute carrier family 7 member 11; GPX4, glutathione peroxidase 4; FSP1, ferroptosis suppressor protein 1; FTH1, ferritin heavy chain; TFR1, transferrin receptor 1; FPN1, ferroportin. Data are presented as mean ± standard error (*n* ≥ 6). Statistical comparison was performed using one-way ANOVA coupled with a Tukey post-hoc analysis, * *p* < 0.05, ** *p* < 0.01, *** *p* < 0.001.

m

l

n


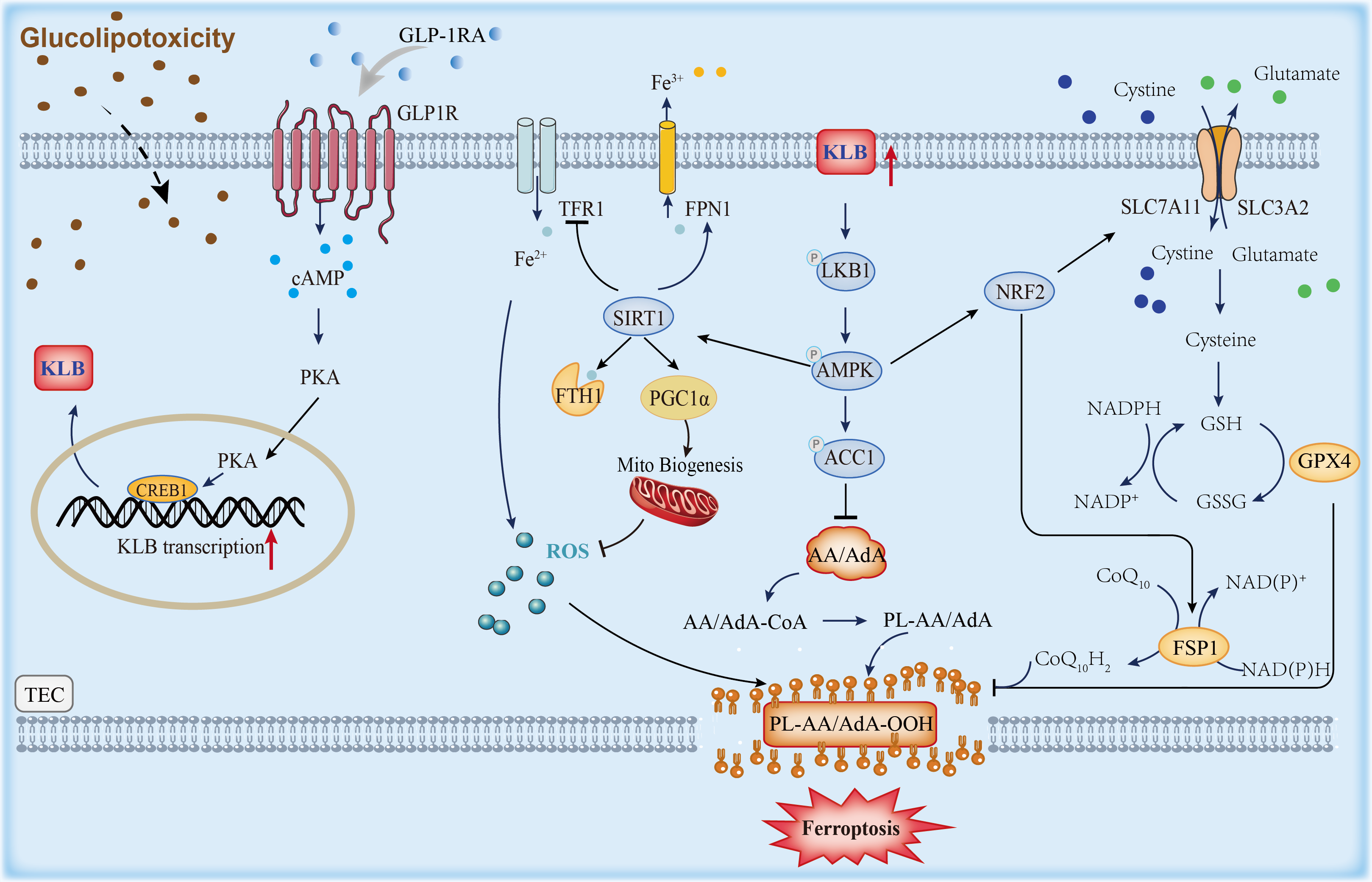


**Figure S9**. Mechanism illustration of Smg upregulates KLB expression and ameliorates diabetic kidney injury through KLB-mediated ferroptosis inhibition. Explanation: GLP-1RA increases KLB expression in the glycolipid condition of HK-2 cells. Specifically, GLP-1RA binds to GLP-1R on the membrane and activates the cAMP/PKA/CREB pathway to up-regulate KLB gene transcription. After the increase of KLB expression, the phosphorylation of ACC1, and the expression of SIRT1 and NRF2 were promoted through the LKB1/AMPKα pathway, which regulated the lipid metabolism, iron metabolism and antioxidant links in the process of ferroptosis, and then inhibited ferroptosis under the condition of high glucose and high lipid. GLP-1RA:glucagon-like peptide-1 receptor agonist; cAMP: cyclic adenosine monophosphate; PKA: Protein kinase A; CREB: cAMP-response element binding protein; TEC: Tubular epithelial cell; KLB, β-Klotho; LKB1, liver kinase beta 1; AMPK, AMP-activated protein kinase; ACC, acetyl-CoA carboxylase; SIRT1, sirtuin 1; NRF2, nuclear factor erythroid 2-related factor 2; SLC7A11, solute carrier family 7 member 11; SLC3A2:solute carrier family 3 member 2; GSSG: Glutathione disulfide; GPX4, glutathione peroxidase 4; NADPH: Nicotinamide adenine dinucleotide phosphate hydrogen; CoQ10: Coenzyme Q10; FSP1, ferroptosis suppressor protein 1; FTH1, ferritin heavy chain; TFR1, transferrin receptor 1; FPN1, ferroportin; AA/AdA: arachidonic acid/adrenic acid; PL-AA/AdA-OOH: phosphatidylethanolamine hydroperoxide containing arachidonic acid or adrenic acid; PGC-1α: proliferator-activated receptor gamma coactivator 1α; ROS: reactive oxygen species.

**Table S1.** Baseline demographic and clinical characteristics of participants. Data are presented as mean ± standard error ( *n* ≥13). Statistical comparison was performed using an unpaired two-tailed Student’s t-test. *p* < 0.05 was considered statistically significant. HbA1c: Hemoglobin A1c; UACR: Urinary Albumin/Creatinine Ratio; NAG: N-acetyl-β-D-glucosaminidase; RBP: Retinol Binding Protein; TRF: Transferrin; β2MG: β2-microglobulin.

| Groups | DKD/Ins ( *n*=15 ) | DKD/Smg ( *n*=13 ) | *P* value |
| --- | --- | --- | --- |
| Age, y | 53.60 ± 8.53 | 50.46 ± 12.18 | 0.432 |
| Male, % | 8.00 ( 53.33 ) | 7.00 ( 53.85 ) | 0.978 |
| HbA1c, % | 7.92 ± 0.56 | 8.23 ± 0.71 | 0.208 |
| Fasting blood glucose, mmol l ^-1^ | 9.33 ± 0.98 | 9.71 ± 0.85 | 0.292 |
| Bodyweight, kg | 69.31 ± 7.58 | 75.70 ± 7.48 | 0.473 |
| BMI, kg m^-2^ | 25.74 ± 2.57 | 26.86 ± 2.11 | 0.222 |
| Waist-to-Hip Ratio | 0.90 ± 0.08 | 0.90 ± 0.08 | 0.873 |
| Systolic BP, mm Hg | 131.33 ± 18.45 | 133.54 ± 11.10 | 0.552 |
| Diastolic BP, mm Hg | 80.07 ± 11.20 | 78.15 ± 8.27 | 0.374 |
| Albumin, g l ^-1^ | 43.69 ± 2.46 | 44.94 ± 3.78 | 0.304 |
| Total protein, g l ^-1^ | 71.83 ± 6.83 | 73.82 ± 4.40 | 0.376 |
| Alanine transaminase, U l ^-1^ | 20.93 ± 4.33 | 26.90 ± 12.26 | 0.089 |
| Aspartate transaminase, U l ^-1^ | 18.89 ± 4.86 | 21.48 ± 4.68 | 0.164 |
| Alkaline phosphatase, U l ^-1^ | 90.66 ± 22.79 | 92.93 ± 32.58 | 0.831 |
| Blood urea nitrogen, μmol l ^-1^ | 9.52 ± 3.67 | 8.35 ± 4.88 | 0.474 |
| Serum creatinine, μmol l ^-1^ | 140.68 ± 21.40 | 145.51 ± 24.09 | 0.579 |
| eGFR, mL min ^-1^/1.73 m^2^ | 44.07 ± 9.37 | 43.28 ± 8.68 | 0.818 |
| Serum uric acid, μmol l ^-1^ | 351.93 ± 83.54 | 333.67 ± 130.00 | 0.658 |
| Triglycerides, mmol l ^-1^ | 2.28 (1.40, 3.52) | 2.35 ± 0.64 | 0.070 |
| Total cholesterol, mmol l ^-1^ | 5.26 ± 1.19 | 4.87 ± 0.83 | 0.202 |
| High density lipoprotein, mmol l ^-1^ | 1.02 ± 0.25 | 0.97 ± 0.22 | 0.053 |
| Low density lipoprotein, mmol l ^-1^ | 3.42 ± 0.82 | 3.20 ± 0.63 | 0.175 |
| UACR, mg g ^-1^ | 876.03(431.20, 1500.03 ) | 898.98 (332.01,2189.00 ) | 0.964 |
| NAG, U l ^-1^ | 16.11 ± 7.44 | 18.54 ± 9.82 | 0.463 |
| RBP, mg l ^-1^ | 7.44 (1.82, 13.48 ) | 7.86 ( 3.64, 26.29 ) | 0.586 |
| TRF, mg l ^-1^ | 21.20 (4.92, 34.37) | 21.14 (14.38, 25.84) | 0.786 |
| β2MG, mg l ^-1^ | 1.15 (0.29, 2.43 ) | 0.73 (0.27, 2.66 ) | 0.928 |
| IgG, mg l ^-1^ | 44.68 (15.08, 84.98) | 24.75 (20.32, 37.47) | 0.201 |
| Oral antidiabetes treatment |  |  |  |
| Biguanides | 12 ( 80.00% ) | 9 ( 69.23% ) | 0.512 |
| α-glucosidase inhibitors | 10 ( 66.67% ) | 8 ( 61.54% ) | 0.778 |
| Sulfonylureas | 1 ( 6.67% ) | 2 ( 15.38% ) | 0.455 |
| Non-sulfonylurea agents | 2 ( 13.33% ) | 2 ( 15.38% ) | 0.877 |
| Thiazolidinediones | 3 ( 20.00% ) | 3 ( 23.08% ) | 0.843 |
| SGLT2 inhibitors | 6 ( 40.00% ) | 5 ( 38.46% ) | 0.934 |

**Table S2.** Key Resources Table

| REAGENT OR RESOURCE | SOURCE | IDENTIFIER |
| --- | --- | --- |
| Antibodies |  |  |
| KLB | Abcam | CAT: Ab106794. RRID: AB_11131597 |
| GPX4 | Abclonal | CAT: A1933. RRID: AB_2763960 |
| LKB1 | ABclonal | CAT: A2122. RRID: AB_2764141 |
| p-LKB1 | ABclonal | CAT: AP0602. RRID: AB_2771578 |
| AMPKα | ABclonal | CAT: A1229. RRID: AB_2759147 |
| p-AMPKα | ABclonal | CAT: AP0432. RRID: AB_2771454 |
| ACC1 | ABclonal | CAT: A15606. RRID: AB_2763012 |
| p-ACC1 | ABclonal | CAT: AP0298. RRID: AB_2770893 |
| Smad2 | ABclonal | CAT: A11498. RRID: AB_2758585 |
| p-Smad2 | ABclonal | CAT: AP0269. RRID: AB_2771542 |
| Smad3 | ABclonal | CAT: A22133. RRID: AB_3083769 |
| p-Smad3 | ABclonal | CAT: AP0727. RRID: AB_2863813 |
| IL-6 | ABclonal | CAT: A11115. RRID: AB_2758413 |
| IL-1β | ABclonal | CAT: A16288. RRID: AB_2769945 |
| E-cad | ABclonal | CAT: A3044. RRID: AB_2764849 |
| Vimentin | ABclonal | CAT: A19607. RRID: AB_2862696 |
| GLP-1R | ABclonal | CAT: A8547. RRID: AB_2769619 |
| GAPDH | ABclonal | CAT: AC002. RRID: AB_2736879 |
| SLC7A11 | Affinity | CAT: DF12509. RRID: AB_2845314 |
| FTH1 | Affinity | CAT: DF6278. RRID: AB_2838244 |
| TFR1 | Affinity | CAT: AF5343. RRID: AB_2837828 |
| FPN1 | Affinity | CAT: DF13561. RRID: AB_2846580 |
| NRF2 | Affinity | CAT: AF0639. RRID: AB_2833793 |
| TNF-α | Affinity | CAT: AF7014. RRID: AB_2835319 |
| NFκB | Affinity | CAT: AF5006. RRID: AB_2834847 |
| p-NFκB | Affinity | CAT: AF2006. RRID: AB_2834435 |
| p-PKA (Thr 197) | Cell Signaling | CAT: 5661. RRID: AB_10707163 |
| FSP1 | Proteintech | CAT: 20886-1-AP. RRID: AB_2878756 |
| SIRT1 | Proteintech | CAT: 13161-1-AP. RRID: AB_10646436 |
| MCP-1 | Proteintech | CAT: 26161-1-AP. RRID: AB_2918100 |
| TGF-β1 | Proteintech | CAT: 21898-1-AP. RRID: AB_2811115 |
| α-SMA | Proteintech | CAT: 14395-1-AP. RRID: AB_2223009 |
| PKA | Proteintech | CAT: 27398-1-AP. RRID: AB_2880861 |
| p-CREB1 (Ser 133) | Proteintech | CAT: 28792-1-AP. RRID: AB_2918203 |
| CREB1 | Proteintech | CAT: 12208-1-AP. RRID: AB_2245417 |
| Anti-rabbit secondary antibody | Simubiotech | CAT: S2001. RRID: AB_3083773 |
| Anti-mouse secondary antibody | Simubiotech | CAT: S2002. RRID: AB_3083774 |
|  |  |  |
| Chemicals, peptides, and recombinant proteins | | |
| ECL | ABclonal | CAT: RM02867 |
| SYBR Green qPCR Mix | ABclonal | CAT: RK21203 |
| Mycoplasma Removal Agent | Beyotime | CAT: C0280S |
| Glucose | Biofroxx | CAT: 1179GR500 |
| Trypsin | Cell Signaling | CAT: 7406S |
| FerroOrange | Dojindo | CAT: F374 |
| Liperfluo | Dojindo | CAT: L248 |
| Mito-FerroGreen | Dojindo | CAT: M489 |
| DMEM culture media | Gibco | CAT: C11885500BT |
| Opti-Mem culture media | Gibco | CAT: 51985091 |
| Semaglutide | Novo Nordisk | N/A |
| Fetal bovine serum | OriCell | CAT: FBSST-01033-500 |
| Erastin | Selleck Chemicals | CAT: S7242 |
| Ferrostatin-1 | Selleck Chemicals | CAT: S7243 |
| RSL3 | Selleck Chemicals | CAT: S8155 |
| DMSO | Sigma-Aldrich | CAT: 472301 |
| Palmitic acid | Sigma-Aldrich | CAT: P5585-10G |
| STZ | Sigma-Aldrich | CAT: S0130 |
| 60 kcal% Fat | Research DIETS | CAT: D12492 |
| Exendin(9-39) amide | MedChemExpress | CAT: HY-P0264 |
| DAPI | Solarbio | CAT: C0065 |
| Penicillin-Streptomycin | Solarbio | CAT: P1400 |
| Phosphate buffered saline (pH7.4) | Solarbio | CAT: P1010 |
| RIPA lysis buffer | Solarbio | CAT: R0010 |
| Sodium citrate buffer (pH 4.5) | Solarbio | CAT: C1013 |
| TRITC | Thermo Fisher Scientific | CAT: T-2769 |
| Lipofectamine 3000 | Thermo Fisher Scientific | CAT: L3000-015 |
| P 3000 | Thermo Fisher Scientific | CAT: L3000-015 |
| DAB | ZSGB-BIO | CAT: ZLI-9018 |
|  |  |  |
| Critical commercial assays | | |
| Mouse TGF-β1 ELISA kit | ABclonal | CAT: RK00057 |
| Mouse IL-1β ELISA kit | ABclonal | CAT: RK00006 |
| Mouse TNF-α ELISA kit | ABclonal | CAT: RK00027 |
| Mouse IL-10 ELISA kit | ABclonal | CAT: RK00016 |
| NAD^+^/NADH assay kit | Beyotime | CAT: S0175 |
| NADP^+^/NADPH assay kit | Beyotime | CAT: S0179 |
| Iron assay kit | BioAssay | CAT: DIFE-250 |
| BCA protein assay kit | Biosharp | CAT: BL521A |
| CCK8 assay kit | Biosharp | CAT: BS350B |
| Human β-klotho ELISA kit | Cusabio | CAT: CSB-EL012378HU |
| Mouse 4-HNE ELISA kit | Fankew | CAT: F9213-B |
| Mouse IL-6 ELISA kit | ML Bio | CAT: YJ003365 |
| Mouse MCP1 ELISA kit | ML Bio | CAT: YJ008852 |
| Human AA ELISA kit | ML Bio | CAT: YJ336692 |
| Human AdA ELISA kit | ML Bio | CAT: YJ004752 |
| Human 4-HNE ELISA kit | ML Bio | CAT: YJ009685 |
| Human cAMP ELISA kit | ML Bio | CAT: YJ006512 |
| HP total RNA kit | Omega Bio-Tek | CAT: R6834 |
| GSH assay kit | Solarbio | CAT: BC1175 |
| MDA assay kit | Solarbio | CAT: BC0025 |
| DCFH-DA ROS assay kit | Solarbio | CAT: D6470 |
| H&E stain kit | Solarbio | CAT: G1120 |
| Masson's trichrome stain kit | Solarbio | CAT: G1340 |
| PAS stain kit | Solarbio | CAT: G1281 |
|  |  |  |
| Oligonucleotides | | |
| KLB siRNA  Sense: 5'-CCAGGUGCUUCAAGCAAUATT-3'  Antisense: 5'-UAUUGCUUGAAGCACCUGGTT-3' | Genepharma | CAT: A01001 |
| Control siRNA  Sense: 5'-UUCUCCGAACGUGUCACGUTT-3'  Antisense: 5'-ACGUGACACGUUCGGAGAATT-3' | Genepharma | CAT: A06001 |
| hKLB-OE  Sense: 5'- - CGCAAATGGGCGGTAGGCGTG -3'  Antisense: 5'-TAGAAGGCACAGTCGAGG-3' | SWS biotechnology | CAT: FC-6213 |
| PCDNA3.1(+)  Sense: 5'-CGCAAATGGGCGGTAGGCGTG-3'  Antisense: 5'-TAGAAGGCACAGTCGAGG-3' | SWS biotechnology | CAT: FV-073 |
| Human-KLB-qPCR-F:  5'-CAAGTGGAAGGGAGTTGGAAGAAGG-3' | Sangon Biotech | N/A |
| Human-KLB-qPCR-R:  5'-TGGAACCATTCGTGCTGCTGAC-3' | Sangon Biotech | N/A |
| Human-CREB1-qPCR-F:  5'-GAGCCGAGAACCAGCAGAGTG-3' | Sangon Biotech | N/A |
| Human-CREB1-qPCR-R:  5'-ACGGTGGGAGCAGATGATGTTG-3' | Sangon Biotech | N/A |
| Human-GAPDH-qPCR-F:  5'-GGCACCGTCAAGGCTGAGAAC-3' | Sangon Biotech | N/A |
| Human-GAPDH-qPCR-R:  5'-GGTGGCAGTGATGGCATGGAC-3' | Sangon Biotech | N/A |
|  |  |  |
| Experimental models: Cell lines/Organisms |  |  |
| HK-2 cells | ATCC | N/A |
| Mouse: C57BL/6J | Huafukang Animal Centre | N/A |
|  |  |  |
| REAGENT or RESOURCE |  |  |
| GraphPad Prism 8.0.2 | GraphPad | RRID: SCR_002798 |
| Image J 1.53a | Schneider et al. | RRID: SCR_003070 |
